# The phylogeny and systematics of Xiphosura

**DOI:** 10.7717/peerj.10431

**Published:** 2020-12-04

**Authors:** James C. Lamsdell

**Affiliations:** Department of Geology and Geography, West Virginia University, Morgantown, WV, United States of America

**Keywords:** Bayesian inference, Horseshoe crabs, Parsimony, Phylogeny, Systematics, Taxonomy, Xiphosura

## Abstract

Xiphosurans are aquatic chelicerates with a fossil record extending into the Early Ordovician and known from a total of 88 described species, four of which are extant. Known for their apparent morphological conservatism, for which they have gained notoriety as supposed ‘living fossils’, recent analyses have demonstrated xiphosurans to have an ecologically diverse evolutionary history, with several groups moving into non-marine environments and developing morphologies markedly different from those of the modern species. The combination of their long evolutionary and complex ecological history along with their paradoxical patterns of morphological stasis in some clades and experimentation among others has resulted in Xiphosura being of particular interest for macroevolutionary study. Phylogenetic analyses have shown the current taxonomic framework for Xiphosura—set out in the *Treatise of Invertebrate Paleontology* in 1955—to be outdated and in need of revision, with several common genera such as *Paleolimulus* Dunbar, 1923 and *Limulitella* Størmer, 1952 acting as wastebasket taxa. Here, an expanded xiphosuran phylogeny is presented, comprising 58 xiphosuran species as part of a 158 taxon chelicerate matrix coded for 259 characters. Analysing the matrix under both Bayesian inference and parsimony optimisation criteria retrieves a concordant tree topology that forms the basis of a genus-level systematic revision of xiphosuran taxonomy. The genera *Euproops* Meek, 1867, *Belinurus* König, 1820, *Paleolimulus*, *Limulitella*, and *Limulus* are demonstrated to be non-monophyletic and the previously synonymized genera *Koenigiella* Raymond, 1944 and *Prestwichianella* Cockerell, 1905 are shown to be valid. In addition, nine new genera (*Andersoniella* gen. nov.*, Macrobelinurus* gen. nov.*,* and *Parabelinurus* gen. nov. in Belinurina; *Norilimulus* gen. nov. in Paleolimulidae; *Batracholimulus* gen. nov. and* Boeotiaspis* gen. nov. in Austrolimulidae; and *Allolimulus* gen. nov., *Keuperlimulus* gen. nov., and *Volanalimulus* gen. nov. in Limulidae) are erected to accommodate xiphosuran species not encompassed by existing genera. One new species, *Volanalimulus madagascarensis* gen. et sp. nov., is also described. Three putative xiphosuran genera—*Elleria* Raymond, 1944, *Archeolimulus* Chlupáč, 1963, and *Drabovaspis* Chlupáč, 1963—are determined to be non-xiphosuran arthropods and as such are removed from Xiphosura. The priority of *Belinurus* König, 1820 over *Bellinurus* Pictet, 1846 is also confirmed. This work is critical for facilitating the study of the xiphosuran fossil record and is the first step in resolving longstanding questions regarding the geographic distribution of the modern horseshoe crab species and whether they truly represent ‘living fossils’. Understanding the long evolutionary history of Xiphosura is vital for interpreting how the modern species may respond to environmental change and in guiding conservation efforts.

## Introduction

**Figure 1:**
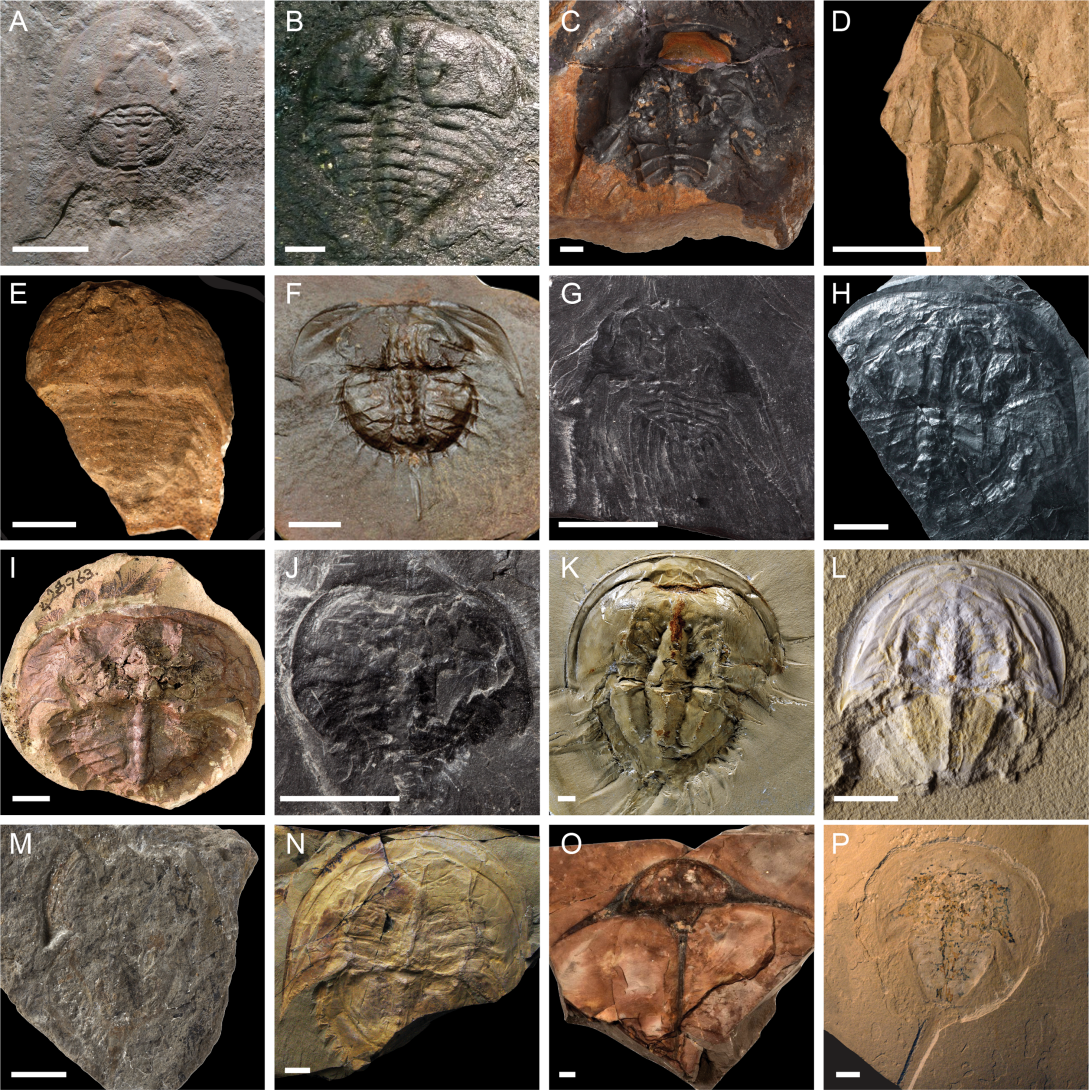
Representatives of the diversity of fossil Xiphosura. (A) *Lunataspis aurora* (MM I-4583 –photo credit: Graham Young); (B) *Kasibelinurus amicorum* (NMS G.2007.271.A, latex cast of AM F68969 –photo credit: James Lamsdell); (C) *Xaniopyramis linseyi* (OUM E.03994 –photo credit: Eliza Howlett); (D) *Paleolimulus signatus* (YPM IP 026324 –photo credit: Jessica Utrup); (E) *Patesia randalli* (FMNH PE.56581 –photo credit: James Lamsdell); (F) *Euproops danae* (YPM IP 000125 –photo credit: Jessica Utrup); (G) *Belinurus carwayensis* (NMW 29.197.G3 –photo credit: Lucy McCobb); (H) *Prestwichianella cambrensis* (NMW 29.198.G1 –photo credit: Lucy McCobb); (I) *Prestwichianella rotundata* (YPM IP 428963 –photo credit: Jessica Utrup); (J) *Belinurus morgani* (BGS GSM49362 –photo credit: Michael Howe); (K) *Mesolimulus walchi* (MNHN.F.A33516 –photo credit: Christian Lemzaouda); (L) *Mesolimulus tafraoutensis* (MSNM i26844 –photo credit: Alessandro Gerassino)*;* (M) *Rolfeia fouldenensis* (NMS G.1984.67.1B –photo credit: Andrew Ross); (N) *Victalimulus mcqueeni* (NMV P22410B –photo credit: Rolf Schmidt); (O) *Austrolimulus fletcheri* (AM F38274 –photo credit: Matthew McCurry); (P) *Tachypleus syriacus* (MSNM i9352 –photo credit: Alessandro Gerassino). Images in (D, F), and (I) made available under a CC0 license courtesy of the Yale Peabody Museum, image in (K ) made available as part of the RECOLNAT (ANR-11-INBS-0004) program, images in (C), (G, H, J), and (M) made available under a CC BY-NC-SA 3.0 license courtesy of the GB3D type fossils database (http://www.3d-fossils.ac.uk/). Scale bars for A–C, F–I, K–P = 10 mm; D, E, J = 5 mm.

**Figure 2:**
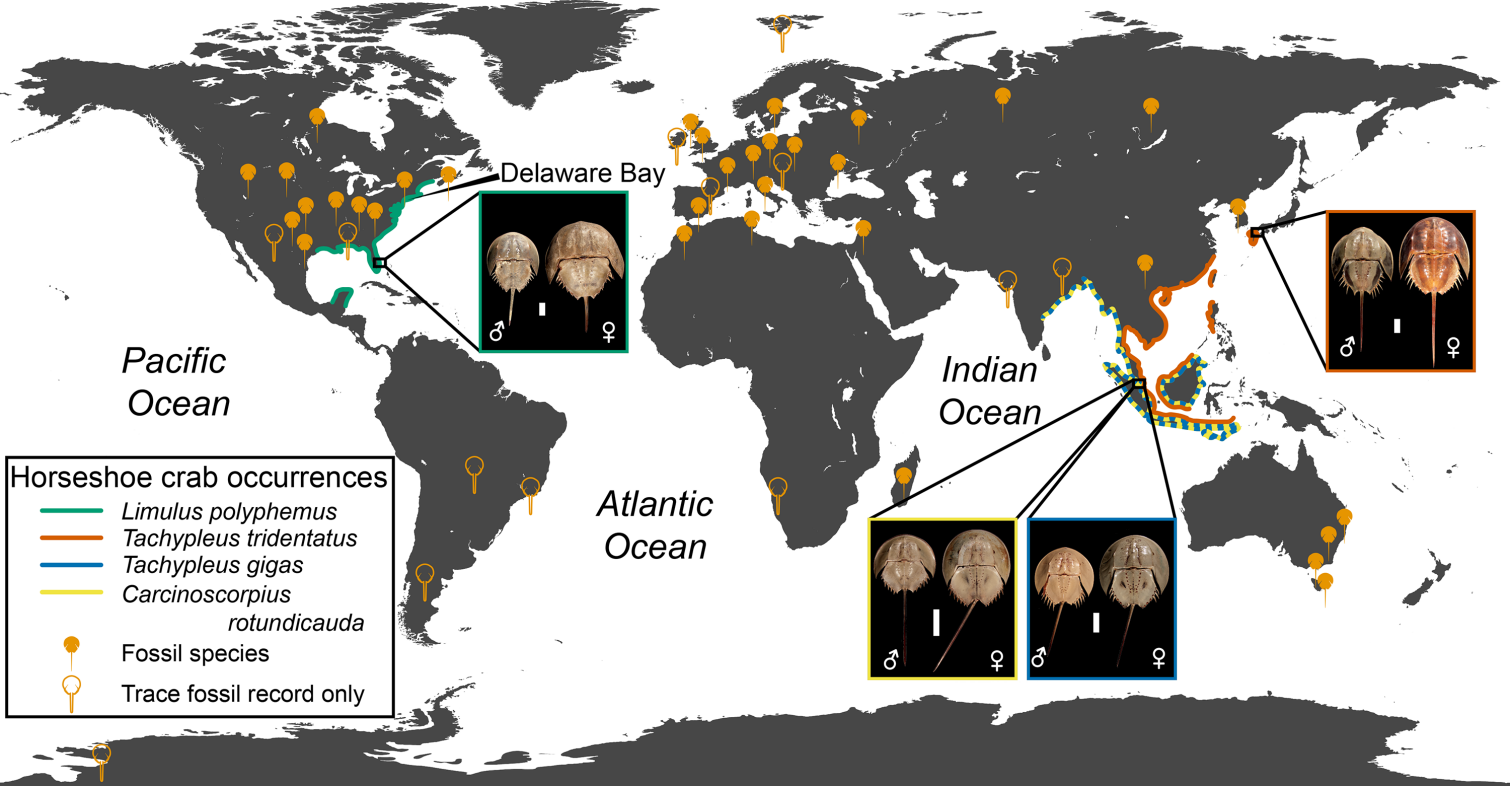
Geographic distribution of modern and fossil horseshoe crabs. Fossil occurrences are derived from Dunlop, Penney & Jekel (2020) for body fossils, with additional trace fossil occurrences from Patel & Shringarpure (1992), Rosa et al. (1994), Pickford (1995), Wignall & Best (2000), Mikuláš & Mertlík (2002), Buta et al. (2005),** Lermen (2006), Chakraborty & Bhattacharya (2012), Hasiotis, Flaig & Jackson (2012), Fernández & Pazos (2013), Naurstad (2014), Lerner & Lucas (2015), Alberti, Fürsich & Pandey (2017), and Mujal et al. (2018). *Limulus polyphemus* is represented by YPM IZ 055605 (male) and YPM IZ 070174 (female), *Carcinoscorpius rotundicauda* by YPM IZ 055595 (male) and YPM IZ 055574 (female), *Tachypleus gigas* by YPM IZ 055578 (male) and YPM IZ 055570 (female), and *Tachypleus tridentatus* by YPM IZ 055581 (male) and YPM IZ 055576 (female). Photo credit for all specimens: James Lamsdell. Scale bars = 50 mm.

Xiphosurans, colloquially known as horseshoe crabs, are a clade of aquatic chelicerates represented by four extant species (Lamsdell, in press) with an evolutionary history stretching back 470 million years to the Ordovician (Rudkin, Young & Nowlan, 2008; Van Roy et al., 2010). Horseshoe crabs are considered archetypal ‘living fossils’ due to their low diversity and apparent morphological conservatism (Fisher, 1984; Fisher, 1990; Kin & Błaźejowski, 2014). However, their fossil record reveals that horseshoe crabs have in the past exhibited a relatively high species diversity (Lamsdell, in press) and a greater variation in both morphology and ecology (Lamsdell, 2016) than their modern representatives (Fig. 1). Although three of the four modern horseshoe crab species are distributed mainly along the coast of Indonesia and the Bay of Bengal, with one species extending into the South and East China Seas, the American horseshoe crab *Limulus polyphemus* (Linnaeus, 1758) is found on the Atlantic coast of North America and the Gulf of Mexico, hinting at a more complex biogeographic history that is borne out by the global distribution of the horseshoe crab fossil record (Fig. 2).

Although there have been several studies on the phylogenetic relationships of the extant horseshoe crab species (Shishikura et al., 1982; Xia, 2000; Kamaruzzaman et al., 2011; Obst et al., 2012; Baek et al., 2014; Periasamy, Ingole & Meena, 2017; Shingate et al., 2020), analyses of fossil horseshoe crab phylogeny have until recently been limited. Anderson & Selden (1997) undertook an analysis of Palaeozoic horseshoe crabs with a focus on synziphosurines, which were at the time considered to be horseshoe crabs with freely articulating body segments, although subsequent analysis has shown the synziphosurines are a polyphyletic collection of euchelicerates that do not resolve within the xiphosuran clade (Lamsdell, 2013; Lamsdell et al., 2015; Selden, Lamsdell & Liu, 2015). More recently, phylogenetic analysis of extant and fossil limuloid xiphosurans suggested that molecular rate estimates for the living species were significantly underestimating their divergence times (Lamsdell & McKenzie, 2015). Subsequent analyses have expanded the taxon sampling to encompass all major xiphosuran clades in order to study the relationship between ecological occupation and morphological disparity (Lamsdell, 2016) and ecological occupation and heterochronic trends (Lamsdell, 2020). These investigations suggested that a number of xiphosuran genera were para- or polyphyletic, as well as inferring major revisions to xiphosuran higher-level taxonomy. In particular, several Permian-Triassic freshwater species were shown to resolve in a distinctive clade with the aberrant *Austrolimulus*, while the genera *Belinurus* and *Euproops* resolved as paraphyletic and the genera *Paleolimulus* and *Limulitella* were revealed to be polyphyletic wastebasket taxa.

Despite the systematic revisions necessitated by the retrieved phylogenetic topologies, no major revision of xiphosuran taxonomy has been undertaken since the publication of the chelicerate *Treatise of Invertebrate Paleontology* volume, within which Størmer (1955) set out the taxonomic framework that was used for the next 60 years. While the higher-level taxonomy of Xiphosura was updated to accommodate the phylogenetic topology (Lamsdell, 2016), no in-depth diagnoses of the higher taxa was attempted, and no diagnostic revision of genera was undertaken. Given that many paleobiological meta-analyses operate at the genus level (Hendricks et al., 2014) and that Xiphosura is a clade of particular interest to evolutionary biologists (Renwick, 1968; Barthel, 1974; Fisher, 1981; Fisher, 1984; Fisher, 1990; Mattei et al., 2010; Faurby et al., 2011; Haug et al., 2012; Kin & Błażejowski, 2014; Moreau et al., 2014; Göpel & Wirkner, 2015; Lamsdell, 2016; Lamsdell, 2020; Lamsdell, in press; Periasamy, Ingole & Meena, 2017; Shingate et al., 2020), it is critical that the lower-level taxonomy of horseshoe crabs also be revised in line with their phylogenetic relationships.

Here, I present a further expanded phylogenetic analysis of Xiphosura and revise the taxonomy of the group down to the genus level. Revised diagnoses are presented for all xiphosuran genera, and a new species of horseshoe crab from the Triassic of Madagascar is named. A taxonomic revision such as this represents a necessary step towards full integration of the extensive xiphosuran fossil record into study of the modern horseshoe crab species, which are themselves under threat from numerous human activities including harvesting as bait for eel and conch fisheries (Bianchini, Sorensen & Winn, 1981; Berkson & Shuster, 1999; Botton et al., 2015) and the biomedical industry (Rudloe, 1983), infringement on their spawning grounds (Nelson et al., 2016; Pati et al., 2017), and the potential for a total loss of breeding grounds due to human engineered stabilization of coastal environments through groins, barriers and bulkheads which will halt the natural landward progression of beach-marsh systems as sea level rises due to global temperature increases (Botton, Loveland & Jacobsen, 1988; Botton, 2001; Berkson et al., 2009; Hsieh & Chen, 2009). Understanding the evolutionary history of lineages can inform us how they have historically respond to extinction pressures and potentially aid us in predicting the responses of modern species to habitat loss and climate change, and could aid in guiding future conservation policy (Dietl et al., 2015; Kosnik & Kowalewski, 2016).

## Materials & Methods

The phylogeny of Xiphosura was analyzed through an expanded version of the latest iteration of the chelicerate character matrix of Lamsdell (2020), derived incrementally from previous analyses of broader euchelicerate relationships (Lamsdell, 2013; Lamsdell, 2016; Lamsdell & McKenzie, 2015; Lamsdell et al., 2015; Selden, Lamsdell & Liu, 2015). The matrix comprises 259 characters coded for 158 taxa and is available in the online MorphoBank database (O’Leary & Kaufman, 2012) under the project code p3497 (accessible from https://morphobank.org/index.php/Projects/ProjectOverview/project_id/3497) as well as in the Supplementary Information. The species considered in this analysis have been studied over the last decade from both direct observation of the specimens and study of high-definition images. Over this time, species within the collections of the Yale Peabody Museum, Chicago Field Museum, American Museum of Natural History, British Museum of Natural History, University of Manchester Geological Museum, Senckenberg Museum in Frankfurt, British Geological Survey in Nottingham, and National Museums of Scotland in Edinburgh were observed directly.

**Figure 3:**
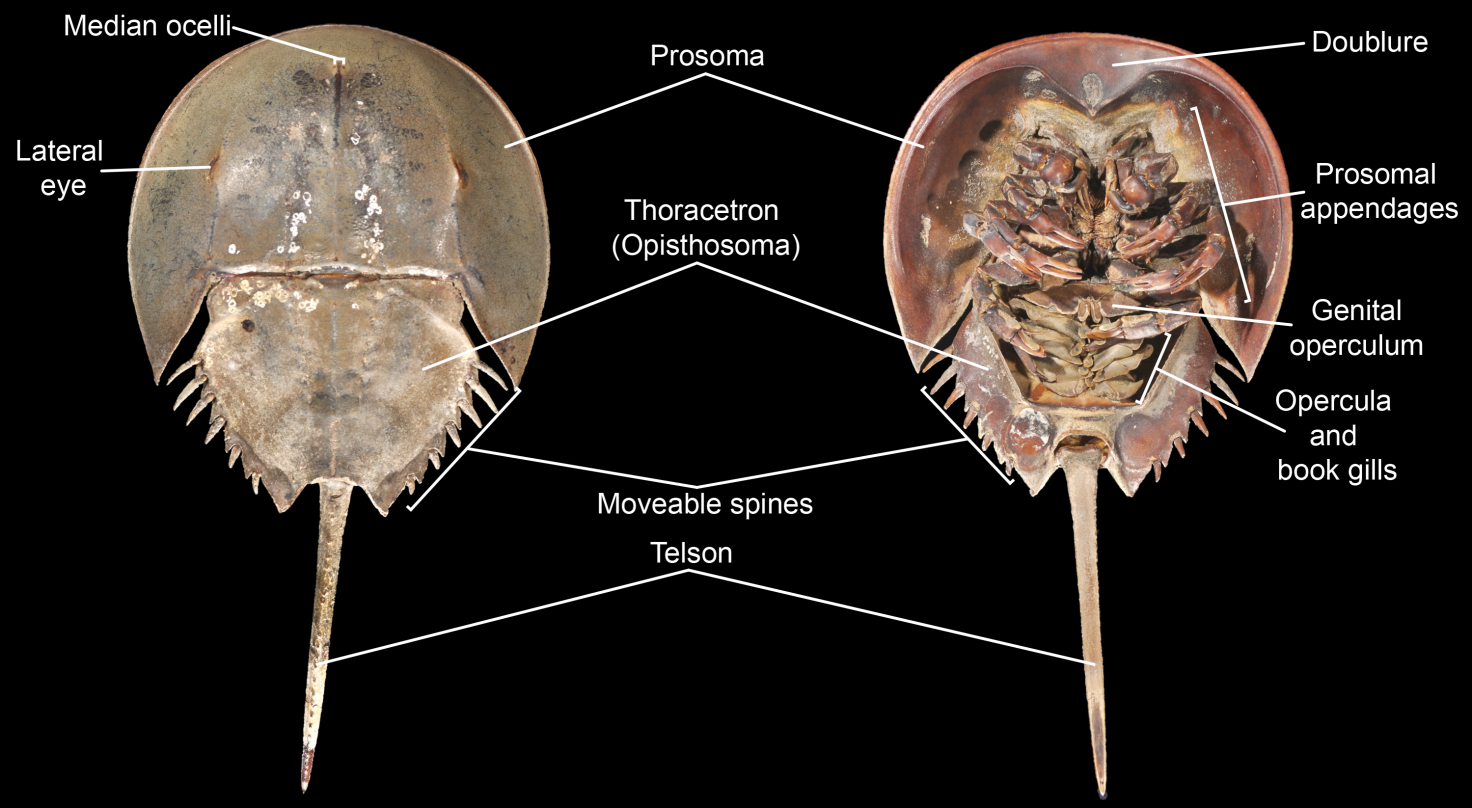
Basic morphological terminology of horseshoe crabs as demonstrated by *Limulus polyphemus* (YPM IZ 070174). Photo credit: James Lamsdell.

Morphological terminology for character definitions follows Selden & Siveter (1987), Lamsdell (2013), Lamsdell (2016) and Lamsdell (2020) –see Fig. 3 for a basic overview of major terms. The number of Xiphosura (*sensu* Lamsdell, 2013, Lamsdell, 2016) sampled within the matrix was increased to 58 through the incorporation of the newly described species *Stilpnocephalus pontebbanus* Selden, Simonetto & Marsiglio, 2019, *Tasmaniolimulus patersoni* Bicknell, 2019 and *Volanalimulus madagascarensis* gen. et sp. nov., and the previously unsampled species *Moravurus rehori* Přibyl, 1967, *Pickettia carteri* (Eller, 1940), *Mesolimulus sibiricus* Ponomarenko, 1985, and *Shpineviolimulus jakovlevi* (Glushenko & Ivanov, 1961). Three new characters (character 16, the lateral eyes being enlarged and bulbous; character 79, pleura of seventh postantennular segment exhibiting curved, lobe-like posterior margin; and character 84, thoracetron lateral margin crenulate) were also added. The recently described species *Xiphosuroides khakassicus* Shpinev & Vasilenko, 2018 was excluded from the analysis due to the species being known only from very early (embryological) instars, the inclusion of juveniles as terminal taxa in phylogenies having been shown to destabilize tree topologies (Lamsdell & Selden, 2013; Lamsdell & Selden, 2015). Two other newly described species, *Sloveniolimulus rudkini* Bicknell et al., 2019 and *Albalimulus bottoni* Bicknell & Pates, 2019, were not included as both species are known from single, poorly preserved specimens lacking diagnostic characters. Three additional species of interest were found to be unsuitable for inclusion in the analysis: *Prolimulus woodwardi* Frič, 1899 is known from several specimens but none preserve details beyond general body outline; *Paleolimulus jurassenensis* Chernyshev, 1933 is known from a single specimen preserving little of the details of the prosoma or thoracetron; and *Limulitella volgensis* Ponomarenko, 1985 is known from multiple specimens that again preserve little detail of the dorsal morphology. Finally, the enigmatic arthropod and possible xiphosurid *Duraznovis gallegoi* Lara et al., 2020 has recently been reinterpreted as the head of a cicadomorph insect (Fu & Huang, 2020), and so was not included in the analysis.

Tree inference was performed as in Lamsdell (2020), via Bayesian inference through Markov-Chain Monte Carlo analyses implemented in MrBayes 3.2.7a (Huelsenbeck & Ronquist, 2001). The dataset was analyzed through four independent runs of 100,000,000 generations and four chains each under the maximum likelihood model for discrete morphological character data with gamma-distributed rate variation among sites (Mkv + Γ: Lewis, 2001). Characters were treated as unordered with equal weighting (Congreve & Lamsdell, 2016). Trees were sampled every 100 generations, resulting in 1,000,000 trees per run, with the first 250,000 sampled trees (25,000,000 generations) of each run discarded as burn-in. The 50% majority rule consensus tree was calculated from the remaining 750,000 sampled trees across all four runs, representing the optimal summary of phylogenetic relationships given the available data (Holder, Sukumaran & Lewis, 2008). The frequency at which a clade occurred among the sampled trees included in the consensus tree was used to calculate posterior probabilities. The matrix was also analyzed under maximum parsimony using TNT (Goloboff, Farris & Nixon, 2008) (made available with the sponsorship of the Willi Hennig Society). The search strategy employed 100,000 random addition sequences with all characters unordered and of equal weight (Congreve & Lamsdell, 2016), each followed by tree bisection-reconnection (TBR) branch swapping (the *mult* command in TNT). Jackknife (Farris et al., 1996), Bootstrap (Felsenstein, 1985) and Bremer (Bremer, 1994) support values were also calculated in TNT. Bootstrapping was performed with 50% resampling for 1,000 repetitions, while jackknifing was performed using simple addition sequence and tree bisection-reconnection branch swapping for 1,000 repetitions with 33% character deletion.

The electronic version of this article in Portable Document Format (PDF) will represent a published work according to the International Commission on Zoological Nomenclature (ICZN), and hence the new names contained in the electronic version are effectively published under that Code from the electronic edition alone. This published work and the nomenclatural acts it contains have been registered in ZooBank, the online registration system for the ICZN. The ZooBank LSIDs (Life Science Identifiers) can be resolved and the associated information viewed through any standard web browser by appending the LSID to the prefix http://zoobank.org/. The LSID for this publication is: urn:lsid:zoobank.org:pub:3653AFDA-318D-4A1D-9E24-4F9F3D30C424. The online version of this work is archived and available from the following digital repositories: PeerJ, PubMed Central and CLOCKSS.

## Results

**Figure 4:**
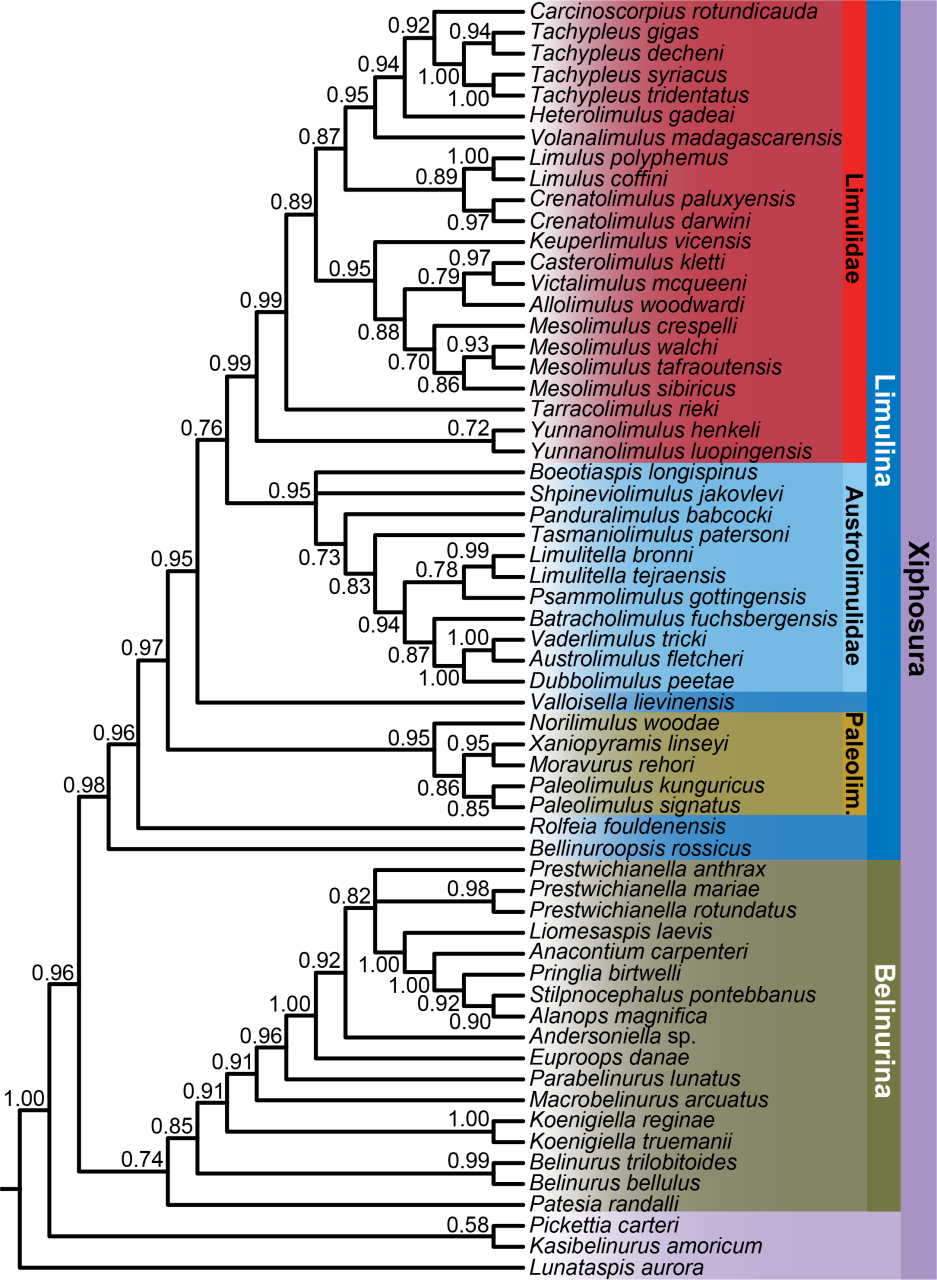
Bayesian phylogeny of xiphosurans, with taxonomic assignments of major clades shown. Bayesian posterior probabilities are shown below each node. As well as the higher taxa labelled in the figure, two important clades are Xiphosurida (comprising Belinurina and Limulina) and Limuloidea (composed of *Valloisella*, Austrolimulidae, and Limulidae). The topology of the strict consensus tree derived from the parsimony analysis is identical to that of the Bayesian phylogeny shown here.

**Figure 5:**
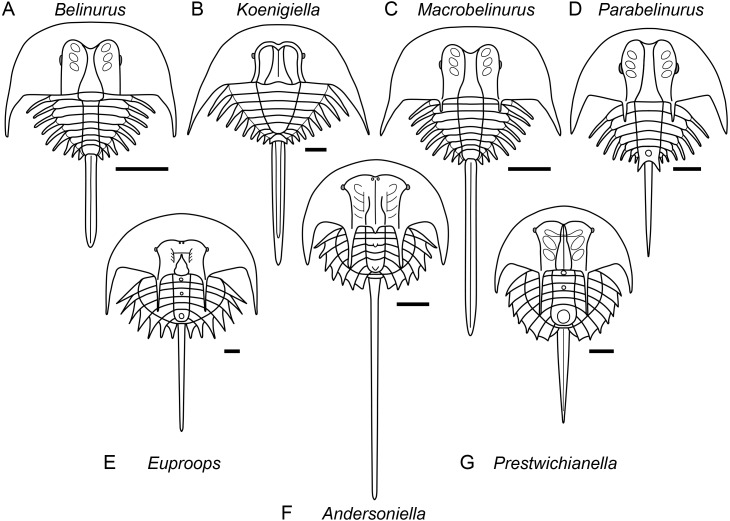
Diagrammatic representation of belinurine genera comprised of species previously assigned to *Belinurus* or *Euproops*. Diagrammatic representation of belinurine genera comprised of species previously assigned to *Belinurus* (A–D) or *Euproops* (E–G). A, *Belinurus*, as represented by *Belinurus bellulus*; B, *Koenigiella*, as represented by *Koenigiella reginae*; C, *Macrobelinurus*, as represented by *Macrobelinurus arcuatus*; D, *Parabelinurus*, as represented by *Parabelinurus lunatus*; E, *Euproops*, as represented by *Euproops danae*; F, *Andersoniella*, as represented by *Andersoniella longispina*; G, *Prestwichianella*, as represented by *Prestwichianella anthrax*.

Analysis of the character matrix resulted in a phylogenetic hypothesis concordant with that of Lamsdell (2020), with Bayesian inference and maximum parsimony once again retrieving a congruent tree topology (Fig. 4). *Lunataspis* (Fig. 1A) resolves as the most basal xiphosuran, befitting its stratigraphic occurrence in the Ordovician, with *Kasibelinurus amoricum* (Fig. 1B) in turn positioned as the sister taxon to Xiphosurida, comprising all other xiphosurans. Xiphosurida is split into two large clades, Belinurina and Limulina. Within Belinurina, the genera *Belinurus* and *Euproops* are once more demonstrated to be paraphyletic, with *Belinurus* grading towards *Euproops*, which in turn grades towards a clade composed of the highly paedomorphic taxa *Liomesaspis*, *Anacontium*, *Pringlia*, and *Alanops*. The non-monophyly of *Belinurus* and *Euproops* is now well-supported and validates the practice of earlier researchers to assign *Belinurus* and *Euproops* species to up to five different genera (Baily, 1859; Cockerell, 1905; Raymond, 1944; Fig. 5).

**Figure 6:**
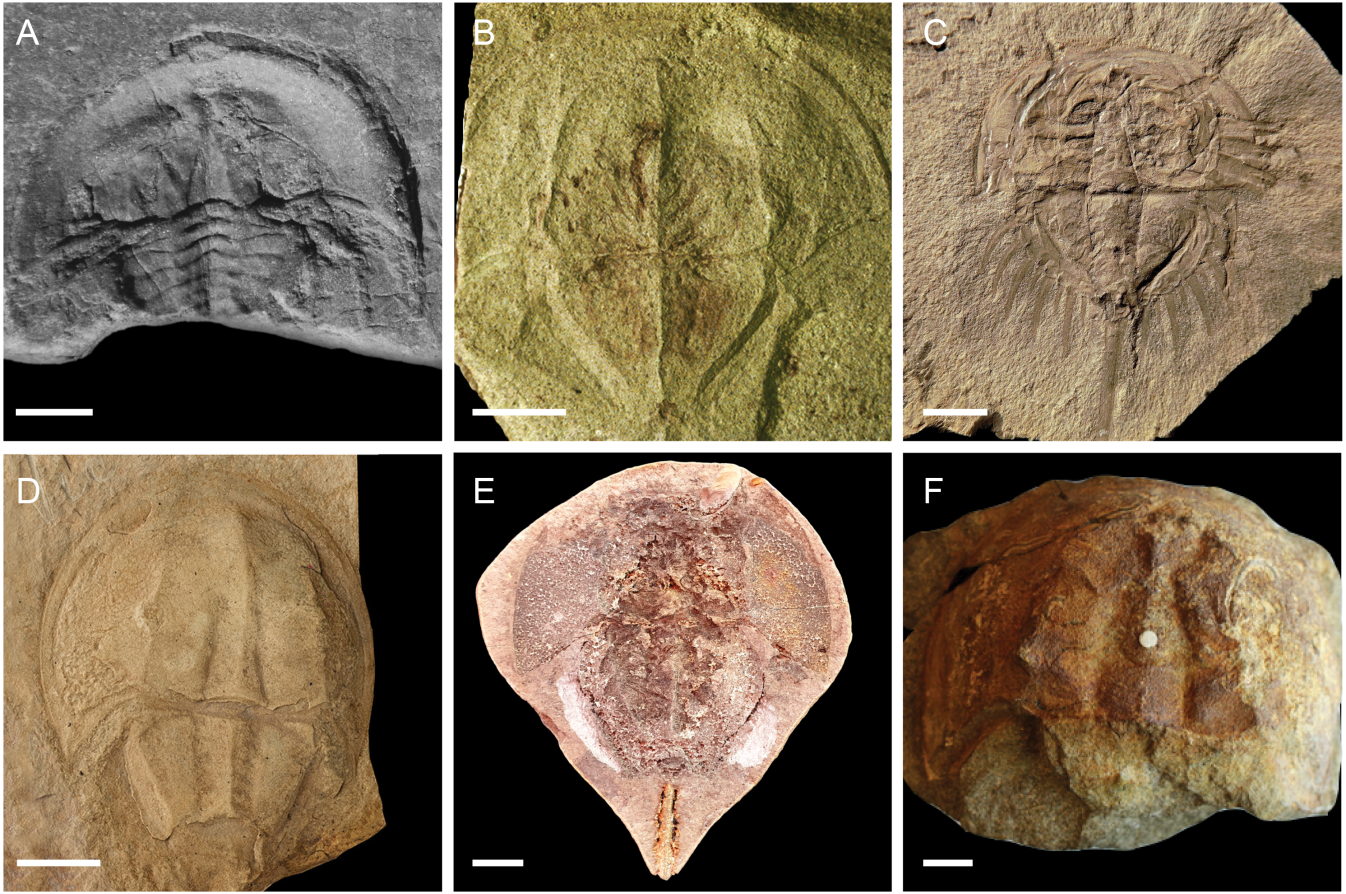
Representatives of newly named xiphosurid genera, excluding belinurines. (A) *Norilimulus woodae* (NSM 005GF045.374 –photo credit: Allan Lerner); (B) *Batracholimulus fuchsbergensis* (SMF VII I 311 –photo credit: Norbert Hauschke) (C) *Boeotiaspis longispinus* (ROM IP 45851 –photo credit: David Rudkin); (D) *Keuperlimulis vicensis* (MAN 8240 –photo credit: Lukáš Laibl); (E) *Volanalimulus madagascarensis* (TUCP Ch.5 –photo credit: Carsten Brauckmann); (F) *Allolimulus woodwardi* (MMUP L.8627 –photo credit: David Gelsthorpe). Images in B and D reproduced from Bicknell & Pates (2020) under a CC BY 4.0 license. Scale bars for A = 2 mm; B = 2 mm; C–F = 10 mm.

Limulina is composed primarily of three clades; Paleolimulidae, Austrolimulidae, and Limulidae. *Tasmaniolimulus* is confirmed as a member of Austrolimulidae, resolving as the sister taxon to a clade comprising the majority of the austrolimulids (*Psammolimulus*, *Dubbolimulus*, *Vaderlimulus*, *Austrolimulus*, ‘*Paleolimulus*’ *fuchsbergensis* (Fig. 6B), and *Limulitella sensu stricto*). The newly described *Volanalimulus madagascarensis* gen. et sp. nov. (Fig. 6E) is placed at the base of the tachypleine limulid clade, which includes the extant *Carcinoscorpius* and *Tachypleus* along with the extinct *Heterolimulus*. *Heterolimulus* is again retrieved as a distinct genus to *Tachypleus* and *Casterolimulus* once again resolves as sister taxon to *Victalimulus* within Limulidae rather than an austrolimulid (following Lamsdell, 2020, contra Lamsdell & McKenzie, 2015; Lamsdell, 2016).

Several limulid genera are confirmed as polyphyletic, corroborating the results of previous analyses (Lamsdell & McKenzie, 2015; Lamsdell, 2016; Lamsdell, 2020). *Paleolimulus*, as historically defined, includes species resolving both within Paleolimulidae and Austrolimulidae. The type species, *Paleolimulus signatus* (Beecher, 1904) (Fig. 1D), and *Paleolimulus kunguricus* Naugolnykh, 2017 comprise the only true members of *Paleolimulus*, with ‘*Paleolimulus*’ *woodae* Lerner, Lucas & Mansky, 2016 (Fig. 6A) representing a distinct genus within Paleolimulidae. ‘*Paleolimulus*’ *longispinus* Schram, 1979 (Fig. 6C), meanwhile, resolves at the base of the austrolimulids and represents another new genus. The genus *Limulitella* resolves as an austrolimulid and contains the type species, *Limulitella bronni* Schimper, 1853, and the recently described *Limulitella tejraensis* Błażejowski et al., 2017. Two other *Limulitella* species resolve within the Limulidae; ‘*Limulitella*’ *henkeli* Fritsch, 1906 as the congeneric sister species to *Yunnanolimulus luopingensis* Zhang et al., 2009, and ‘*Limulitella*’ *vicensis* Bleicher, 1897 (Fig. 6D) representing a new genus closely related to *Mesolimulus*, *Victalimulus*, and *Casterolimulus*. *Patesia randalli* (Beecher, 1902), previously considered a species of *Kasibelinurus*, resolves as a distinct genus at the base of the Belinurina. Finally, *Limulus* is shown to be polyphyletic, with several fossil species resolving outside of the clade including the living type species, *Limulus polyphemus* (Linnaeus, 1758). ‘*Limulus*’ *darwini* Kin & Błażejowski, 2014 is congeneric with *Crenatolimulus paluxyensis* Feldmann et al., 2011, while ‘*Limulus*’ *woodwardi* Watson, 1909 (Fig. 6F) resolves as a novel genus with close affinities to *Victalimulus* and *Casterolimulus*. *Limulus coffini* Reeside & Harris, 1952 remains as the only extinct species demonstrably assignable to the genus.

The consistency of the tree topology over five years of analyses along with the concordant tree topologies retrieved via Bayesian inference and parsimony optimality criteria provide a strong rationale for a systematic revision of the genus-level taxonomy of Xiphosura, building upon previous higher-level taxonomic revisions (Lamsdell & McKenzie, 2015; Lamsdell, 2016).

### Systematic Palaeontology

**Table:** 

Subphylum CHELICERATA Heymons, 1901
Class XIPHOSURA Latreille, 1802
[=MEROSTOMATA Dana, 1852]

*Included taxa*. *Lunataspis* Rudkin, Young & Nowlan, 2008; Kasibelinuridae Pickett, 1993; Xiphosurida Latreille, 1802.

*Distribution.* Ordovician–recent; worldwide. Fossil representatives known from every major continent, including fossil trackways in Antarctica (see Fig. 2).

*Emended diagnosis*. Chelicerata with unfused appendage VII; cardiac lobe extending anteriorly beyond the posterior half of carapace; vaulted prosomal shield covering appendages dorsally and laterally; width of opisthosomal axis equal to that of cardiac lobe; segments VIII–XIV fused into thoracetron (after Lamsdell, 2016).

*Remarks*. *Elleria* Raymond, 1944, comprising the single species *Elleria morani* (Eller, 1938a), is known from a single incomplete specimen interpreted as a partial thoracetron. The specimen comes from the Upper Devonian of the marine Venango Formation and so could be important as one of the few Devonian xiphosurans known; however, the ring-like morphology of the axial region, complete with axial nodes, and the curvature of the tergite boundaries are not comparable to any known xiphosuran. Instead, *Elleria morani* most likely represents a damaged trilobite pygidium, and as such it is here removed from Xiphosura. Other putative xiphosurans from the Middle Ordovician of the Czech Republic, *Archeolimulus hanusi* Chlupáč, 1963 and *Drabovaspis complexa* Chlupáč, 1963, are bradoriid arthropods and are also excluded from Xiphosura.

**Table:** 

*Lunataspis* Rudkin, Young & Nowlan, 2008
(Fig. 1A)

*Type and only species*. *Lunataspis aurora* Rudkin, Young & Nowlan, 2008.

*Distribution.* Ordovician; Canada.

*Emended diagnosis*. Xiphosura with lunate prosomal shield; ophthalmic ridges weak, flanking low cardiac lobe; posterior margin of prosomal shield bowed forward in shallow, blunt V-shaped embayment between broad-based genal spines; subpentagonal thoracetron composed of seven tergites; metasoma composed of three tergites; telson lanceolate, depressed triangular in cross section.

**Table:** 

Family KASIBELINURIDAE Pickett, 1993

*Type genus. Kasibelinurus* Pickett, 1993.

*Included genus*. *Pickettia* Bicknell, Lustri & Brougham, 2019.

*Distribution*. Devonian; Australia and United States.

*Emended diagnosis*. Xiphosura with triangular thoracetron, narrowing evenly towards the posterior and terminating in enlarged pretelson.

*Kasibelinurus* Pickett, 1993

(Fig. 1B)

*Type and only species*. *Kasibelinurus amicorum* Pickett, 1993.

*Distribution*. Devonian; Australia.

*Emended diagnosis*. Kasibelinurid with broad prosomal shield possessing short genal spines; cardiac lobe defined by strongly impressed cardiac furrow; thoracetron triangular, narrowing evenly towards the posterior; thoracetron lacking axial nodes.

*Pickettia* Bicknell, Lustri & Brougham, 2019

*Type and only species*. *Bellinurus carteri* Eller, 1940.

*Distribution*. Devonian; United States.

*Emended diagnosis*. Kasibelinurid with broad prosomal shield possessing short genal spines; cardiac lobe defined by strongly impressed cardiac furrow; thoracetron triangular, narrowing evenly towards the posterior; thoracetron axis equal in width to ophthalmic ridges; thoracetron lacking axial nodes.

**Table:** 

Order XIPHOSURIDA Latreille, 1802

*Included taxa*. Belinurina Zittel in Zittel & Eastman, 1913; Limulina Richter & Richter, 1929.

*Distribution*. Devonian–recent; worldwide.

*Emended diagnosis*. Xiphosura with sagittal keel on prosomal shield; postabdomen comprising a single segment (after Lamsdell, 2016).

Suborder BELINURINA Zittel in Zittel & Eastman, 1913

*Included taxa*. Belinuridae Zittel in Zittel & Eastman, 1913.

*Distribution*. Devonian–Permian; Canada, Czech Republic, France, Germany, Italy, Korea, Poland, Russia, Ukraine, United Kingdom, and United States.

*Emended diagnosis*. Xiphosurida with trunk doublure dorsally delineated by furrow; tergopleurae present on posterior tergites; thoracetron lacking moveable spines; tergites expressed dorsally on thoracetron (after Lamsdell, 2016).

**Table:** 

Family BELINURIDAE Zittel in Zittel & Eastman, 1913
[=EUPROOPIDAE Eller, 1938b; =LIOMESASPIDAE Raymond, 1944]

*Type genus*. *Belinurus* König, 1820 [=*Bellinurus* Pictet, 1846; =*Steropis* Baily, 1859].

*Included genera*. *Alanops* Racheboeuf, Vannier & Anderson, 2002; *Anacontium* Raymond, 1944; *Andersoniella* gen. nov.; *Euproops* Meek, 1867; *Koenigiella* Raymond, 1944; *Liomesaspis* Raymond, 1944; *Macrobelinurus* gen. nov.; *Parabelinurus* gen. nov.; *Patesia* Bicknell & Smith, 2020; *Prestwichianella* Cockerell, 1905 [=*Prestwichia* Woodward, 1867]; *Pringlia* Raymond, 1944 [=*Palatinaspis* Malz & Poschmann, 1993]; *Prolimulus* Frič, 1899; *Stilpnocephalus* Selden, Simonetto & Marsiglio, 2019; *Xiphosuroides* Shpinev & Vasilenko, 2018.

*Distribution*. Devonian–Permian; Canada, Czech Republic, France, Germany, Italy, Korea, Poland, Russia, Ukraine, United Kingdom, and United States.

*Emended diagnosis*. As for Belinurina.

*Remarks*. The genera *Belinurus* and *Euproops*, as have been defined over the past few decades, are paraphyletic. Redefining both *Belinurus* and *Euproops* to be monophyletic validates a number of previously synonymised genera, with *Prestwichianella* and the new genus *Andersoniella* accommodating species with a prior assignment to *Euproops* while *Koenigiella* accommodates species that had been placed within *Belinurus*. Two new genera, *Macrobelinurus* and *Parabelinurus*, incorporate the remainder of the former *Belinurus* species. Conversely, the genus *Xiphosuroides* is most likely a synonym of one of the other belinurine genera, however as it is currently only known from embryological instars and does not co-occur with any other belinurine genera it is currently impossible to determine to which genus *Xiphosuroides khakassicus* belongs.

*Alanops* Racheboeuf, Vannier & Anderson, 2002

*Type and only species*. *Alanops magnificus* Racheboeuf, Vannier & Anderson, 2002.

*Distribution*. Carboniferous; France.

*Emended diagnosis*. Belinurid with subhemispherical prosomal shield lacking ophthalmic ridges and ophthalmic spines; lateral eyes located in antemesial position on the prosomal shield; cardiac lobe poorly differentiated, posteriorly bound by shallow furrows, effaced anteriorly; lacking sagittal keel on prosomal shield; genal spines reduced to small cornua; thoracetron subtriangular, strongly vaulted; tergite boundaries exhibiting no lateral expression; opisthosomal axis displaying four segments, with conical opisthosomal boss posteriorly; apodemal pits present on thoracetron; thoracetron lacking tergopleural fixed spines; trunk doublure not dorsally delineated by furrow; telson long, styliform.

*Anacontium* Raymond, 1944

*Type and only species*. *Anacontium carpenteri* Raymond, 1944 [=*Anacontium brevis* Raymond, 1944]*.*

*Distribution.* Permian; United States.

*Emended diagnosis*. Belinurid with lateral eyes located in antemesial position on the prosomal shield; ophthalmic ridges bowing axially posterior to the lateral eyes; lacking sagittal keel on prosomal shield; cardiac lobe effaced anteriorly; genal spines reduced to small cornua; tergite boundaries exhibiting no lateral expression.

*Andersoniella* gen. nov.

*Type species. Euproops longispina* Packard, 1885.

*Included species. Andersoniella* sp.

*Etymology.* Named for Lyall I. Anderson, who revitalised fossil horseshoe crab research in the 1990s and made invaluable contributions towards resolving the taxonomy of *Euproops* species.

*Distribution*. Carboniferous; Germany and United States.

*Diagnosis*. Belinurid with lateral eyes located in antemesial position on the prosomal shield; ophthalmic ridges bowing axially posterior to the lateral eyes; cardiac lobe bordered by dorsal furrows; ophthalmic spines positioned at posterior of ophthalmic ridges, elongated and extending over thoracetron; tergopleural fixed spines expanded proximally, forming opisthosomal flange; conical opisthosomal boss present.

*Remarks*. *Andersoniella* sp., the undescribed species referred to as ‘piesproops’ (Haug et al., 2012; Haug & Rötzer, 2018; Haug & Haug, 2020), resolves as a genus distinct to either *Euproops* or *Prestwichianella*. *Andersoniella longispina* shares the same combination of characters as ‘piesproops’ that differentiate *Andersoniella* from *Euproops* and *Prestwichianella*, specifically the antemesial position of the eyes combined with the pleural spines extending beyond the opisthosomal flange (Fig. 5F), and is selected as type species given the ‘piesproops’ has not yet received a formal name and description.

*Belinurus* König, 1820

[=*Bellinurus* Pictet, 1846; =*Steropis* Baily, 1859]

(Figs. 1G,  1J)

*Type species*. *Belinurus bellulus* König, 1820.

*Included species. Belinurus carwayensis* Dix & Pringle, 1929; *Belinurus concinnus* Dix & Pringle, 1929; *Belinurus grandaevus* Jones & Woodward, 1899; *Belinurus kiltorkensis* Baily, 1969; *Belinurus morgani* Dix & Pringle, 1930; *Belinurus pustulosus* Dix & Pringle, 1929; *Belinurus silesiacus* (Roemer, 1883); *Belinurus sustai* (Prantl & Přıibyl 1956); *Belinurus trechmanni* Woodward, 1918; *Belinurus trilobitoides* (Buckland, 1837).

*Distribution.* Carboniferous; Canada, Czech Republic, Germany, and United Kingdom.

*Emended diagnosis*. Belinurid with axis of first thoracetron tergite medially inflated; thoracetron ovoid to semi-circular in outline; thoracetron fixed tergopleural spines elongate, needle-like.

*Remarks*. The taxonomic priority of *Belinurus* and its misspelling *Bellinurus* has been in flux for almost 200 years. Recently, Haug & Haug (2020) argued that the assumption by Morris (1980) that *Belinurus* was proposed in an unpublished monograph by König and that *Bellinurus* Pictet, 1846 had priority is mistaken, and that König’s monograph was published prior to the work of Pictet. Baily (1863) confirms that König’s monograph was indeed published in 1820, and so the name *Belinurus* König, 1820 clearly has priority. As such, the correct spelling of the family containing *Belinurus* is Belinuridae, as determined by Article 35.4.1 of the International Code of Zoological Nomenclature (International Commission on Zoological Nomenclature, 1999).

Baily (1859) proposed the new genus *Steropis* to accommodate the newly described species ‘*Steropis*’ *arcuatus* along with the existing species *Belinurus trilobitoides*, ‘*Limulus*’ *anthrax*, and ‘*Limulus*’ *rotundus* but assigned no types species. Morris (1980) subsequently assigned *Belinurus trilobitoides* as the type, following Article 69.1 of the International Code of Zoological Nomenclature (International Commission on Zoological Nomenclature, 1999).

*Euproops* Meek, 1867

(Fig. 1F)

*Type and only species*. *Bellinurus danae* Meek & Worthen, 1865 [=*Euproops amiae* Woodward, 1918; =*Euproops darrahi* Raymond, 1944; =*Euproops graigolae* Dix & Pringle, 1929; =*Euproops gwenti* Dix & Pringle, 1929; =*Euproops islwyni* Dix & Pringle, 1929; =*Euproops kilmersdonensis* Ambrose & Romano, 1972; =*Euproops laevicula* Raymond, 1944; =*Euproops meeki* Dix & Pringle, 1929; =*Euproops nitida* Dix & Pringle, 1929; =*Euproops packardi* Willard & Jones, 1935; =*Prestwichia (Euproops) scheeleana* Ebert, 1892; =*Euproops thompsoni* Raymond, 1944; =*Prestwichianella zalesskii* Chernyshev, 1927].

*Distribution.* Carboniferous; Russia, Ukraine, and United States.

*Emended diagnosis*. Belinurid with ophthalmic ridges bowing axially posterior to the lateral eyes; cardiac lobe bordered by dorsal furrows; ophthalmic spines positioned at posterior of ophthalmic ridges, elongated and extending over thoracetron; tergopleural fixed spines expanded proximally, forming opisthosomal flange; conical opisthosomal boss present.

*Remarks*. *Euproops* was the most speciose xiphosuran genus, however a dozen species have since been shown to be synonyms of the type species *Euproops danae* (Anderson, 1994; Haug et al., 2012), and the majority of the remaining species should be assigned to the genus *Prestwichianella* (Fig. 5E). *Euproops* now comprises only *Euproops danae*, a cosmopolitan species known from multiple localities across Europe and North America.

*Koenigiella* Raymond, 1944

*Type species*. *Bellinurus reginae* Baily, 1863.

*Included species. Koenigiella baldwini* (Woodward, 1907); *Koenigiella koenigianus* (Woodward, 1872); *Koenigiella longicaudatus* (Woodward, 1907); *Koenigiella truemani* (Dix & Pringle, 1929) .

*Distribution.* Carboniferous; Germany, Poland, and United Kingdom.

*Diagnosis*. Belinurid with ophthalmic ridges slightly bowing axially posterior to the lateral eyes; genal spines drawn out, equal in length to thoracetron; thoracetron axis broad, equal in width to ophthalmic ridges; thoracetron subtriangular in outline; thoracetron fixed tergopleural spines elongate, needle-like.

*Remarks*. *Koenigiella* represents the other main clade within the ‘*Belinurus*’ grade belinurines, comprising belinurines lacking ophthalmic spines with a subtriangular thoracetron (Fig. 5B).

*Liomesaspis* Raymond, 1944

*Type and only species*. *Liomesaspis laevis* Raymond, 1944 [=*Palatinaspis beimbaueri* Malz & Poschmann, 1993; =*Pringlia bispinosa* Raymond, 1944; =*Pringlia demaisterei* Vandenberghe, 1960; = *Pringlia fritschi* Remy & Remy, 1959; =*Pringlia leonardensis* Tasch, 1961].

*Distribution.* Carboniferous–Permian; France, Germany, United Kingdom, and United States.

*Emended diagnosis*. Belinurid with rounded prosomal shield; lateral eyes located in antemesial position on the prosomal shield; ophthalmic ridges bowing axially posterior to the lateral eyes; cardiac lobe effaced anteriorly; genal spines reduced to small cornua; ophthalmic spines positioned at posterior of ophthalmic ridges, elongated and extending over thoracetron; tergite boundaries exhibiting no lateral expression; apodemal pits present on thoracetron; thoracetron lacking tergopleural fixed spines; conical opisthosomal boss present.

*Macrobelinurus* gen. nov.

*Type and only species*. *Steropis arcuatus* Baily, 1859.

*Etymology*. *Macro*, meaning long, affixed to *Belinurus* (meaning needle-tailed), in reference to the extreme length of the telson and the morphological similarity of the genus to *Belinurus* König, 1820.

*Distribution.* Carboniferous; United Kingdom.

*Diagnosis*. Belinurid with ophthalmic ridges bowing axially posterior to the lateral eyes; ophthalmic spines positioned at posterior of ophthalmic ridges; thoracetron fixed tergopleural spines elongate, needle-like.

*Remarks. Macrobelinurus arcuatus* is an isolated species that resolves intermediate between the *‘Belinurus*’ grade belinurines and species showing greater affinity to the ‘*Euproops*’ morphotype. *Macrobelinurus* retains the narrow, elongate tergopleural spines and does not possess an opisthosomal boss, but exhibits the axial bowing of the ophthalmic ridges and development of ophthalmic spines characteristic of ‘*Euproops*’ grade belinurines (Fig. 5C).

*Parabelinurus* gen. nov.

*Type species*. *Enthomolithus lunatus* Martin, 1809.

*Included species. Parabelinurus iswariensis* (Chernyshev, 1928); *Parabelinurus lacoei* (Packard, 1885); *Parabelinurus metschetensis* (Chernyshev, 1928); *Parabelinurus stepanovi* (Chernyshev, 1928).

*Etymology.* From the Greek *παρα* (similar) and *Belinurus* due to its close similarities to the genus *Belinurus* König, 1820.

*Distribution.* Carboniferous; Russia, Ukraine, and United States.

*Diagnosis*. Belinurid with ophthalmic ridges bowing axially posterior to the lateral eyes; ophthalmic spines positioned at posterior of ophthalmic ridges; thoracetron fixed tergopleural spines elongate, needle-like; conical opisthosomal boss present; terminal tergopleural projections fused directly to opisthosomal boss.

*Remarks*. *Parabelinurus* comprises species previously assigned to *Belinurus* that are closest morphologically to the ‘*Euproops*’ grade belinurines, possessing an opisthosomal boss and ophthalmic spines (Fig. 5D). The combination of these characteristics with elongate, needle-like tergopleural spines, axially bowing ophthalmic ridges, and ophthalmic spines mark these species as comprising a distinct genus within Belinurina. The thoracetron is also markedly circular in outline when compared to *Macrobelinurus*, *Belinurus*, and *Koenigiella*. The fusion of the terminal tergopleural projections to the opisthosomal boss potentially serves as a synapomorphy for the genus.

*Patesia* Bicknell & Pates, 2020

(Fig. 1E)

*Type and only species*. *Prestwichia randalli* Beecher, 1902 [=*Belinurus alleganyensis* Eller, 1938b]*.*

*Distribution.* Carboniferous; United States.

*Emended diagnosis*. Belinurid with thoracetron fixed tergopleural spines elongate, needle-like; intratergal ridges present on thoracetron segments; pretelson comprised of two segments.

*Remarks*. *Patesia randalli* has been recognized as representing a distinct genus of xiphosurid for almost a decade (Lamsdell, Xue & Selden, 2013). Recently, the species was redescribed as a stem xiphosurid having diverged prior to the split between Belinurina and Limulina ( Bicknell & Smith, 2020). The diagnosis given for the genus, based solely on the type material, interpreted a number of characteristics (such as fixed pleural spines) as absent although the corresponding regions are not preserved on the material studied. Additional material in the process of being described confirms *Patesia* as a member of Belinurina and forms the basis for the emended diagnosis.

*Prestwichianella* Cockerell, 1905

[=*Prestwichia* Woodward, 1867 (preoccupied)]

(Figs. 1H, 1I )

*Type species*. *Limulus anthrax* Prestwich, 1840.

*Included species. Prestwichianella bifida* (Siegfried, 1972); *Prestwichianella cambrensis* (Dix & Pringle, 1929); *Prestwichianella rotundata* (Prestwich, 1840); *Prestwichianella mariae* (Crônier & Courville, 2005); *Prestwichianella* (?) *orientalis* (Kobayashi, 1933).

*Distribution.* Carboniferous; France, Germany, Korea, Poland, and United Kingdom.

*Emended diagnosis*. Belinurid with lateral eyes located in antemesial position on the prosomal shield; ophthalmic ridges bowing axially posterior to the lateral eyes; ophthalmic spines positioned at posterior of ophthalmic ridges, elongated and extending over thoracetron; cardiac lobe with quadrate anterior expansion; tergopleural fixed spines expanded proximally, forming opisthosomal flange; pleural spines reduced beyond opisthosomal flange; conical opisthosomal boss present.

*Remarks*. *Prestrichianella* is comprised of species previously included within *Euproops* that have reduced pleural spines and laterals eyes located antemesially on the prosomal shield and plot phylogenetically closest to the highly paedomorphic belinurines such as *Alanops* and *Pringlia* (Fig. 5G).

*Pringlia* Raymond, 1944

*Type and only species*. *Prestwichia birtwelli* Woodward, 1872.

*Distribution.* Carboniferous; United Kingdom.

*Emended diagnosis*. Belinurid with lateral eyes located in antemesial position on the prosomal shield; lacking sagittal keel on prosomal shield; lacking ophthalmic ridges; cardiac lobe effaced anteriorly; genal spines reduced to small cornua; tergite boundaries exhibiting no lateral expression; apodemal pits present on thoracetron; thoracetron lacking tergopleural fixed spines; trunk doublure not dorsally delineated by furrow; conical opisthosomal boss present.

*Remarks*. *Pringlia* shows strong similarities to *Prolimulus* Frič, 1899, and the two genera may be synonyms, in which case the genus *Prolimulus* would have priority. However, the available material of *Prolimulus* is too incomplete to warrant a broader taxonomic revision at this time.

*Prolimulus* Frič, 1899

*Type and only species*. *Prolimulus woodwardi* Frič, 1899.

*Distribution.* Carboniferous; Czech Republic.

*Emended diagnosis*. Belinurid with genal spines reduced; tergite boundaries exhibiting no lateral expression; thoracetron lacking tergopleural fixed spines.

*Remarks*. The available specimens of *Prolimulus* are poorly preserved, and express little in the way of characteristics other than a general round outline to the prosoma and thoracetron and a lack of genal and pleural spines. The available material appears to show a strong affinity to *Alanops and Pringlia*, and there could be an argument for synonymising *Prolimulus* with one of these genera. Lacking more complete material of *Prolimulus*, however, it is considered best to currently retain all three as valid genera within Belinuridae.

*Stilpnocephalus* Selden, Simonetto & Marsiglio, 2019

*Type and only species*. *Stilpnocephalus pontebbanus* Selden, Simonetto & Marsiglio, 2019.

*Distribution.* Carboniferous; Italy.

*Emended diagnosis*. Belinurid with large, highly vaulted, strongly effaced prosomal shield, lacking ophthalmic ridges, genal spines, and ophthalmic spines.

*Xiphosuroides* Shpinev & Vasilenko, 2018

*Type and only species*. *Xiphosuroides khakassicus* Shpinev & Vasilenko, 2018.

*Distribution.* Carboniferous; Russia.

*Emended diagnosis*. Belinurid with embryonic prosomal shield rounded pentagonal in shape and elongated genal spines; embryonic cardiac lobe narrow; embryonic thoracetron with narrow axis and elongated posterior pleural spines.

*Remarks*. *Xiphosuroides*, known only from embryonic individuals, is most likely a junior synonym of one of the other belinurid taxa. However, *Xiphosuroides* is the only xiphosurid known from its type locality, and given the lack of embryonic individuals known from any other belinurine genus it would be imprudent to synonymise *Xiphosuroides* at this time, and so it is retained here as Belinurina *incertae sedis*.

**Table:** 

Suborder LIMULINA Richter & Richter, 1929

*Included taxa*. *Bellinuroopsis* Chernyshev, 1933 [=*Neobelinuropsis* Eller, 1938a]; Limuloidea Zittel, 1885; Paleolimulidae Raymond, 1944; Rolfeiidae Selden & Siveter, 1987.

*Distribution.* Devonian–recent; worldwide.

*Emended diagnosis*. Xiphosurida with the tergites of somites XIV–XV fused; articulating flange present on lateral region of prosomal/opisthosomal joint (after Lamsdell, 2016).

*Bellinuroopsis* Chernyshev, 1933

[= *Neobelinuropsis* Eller, 1938a]

*Type and only species*. *Bellinuroopsis rossicus* Chernyshev, 1933.

*Distribution.* Devonian; Russia.

*Emended diagnosis*. Limuline with wedge-shaped cardiac lobe; thoracetron rounded, composed of eight segments with pleural spines; transverse ridge nodes present on thoracetron.

Family ROLFEIIDAE Selden & Siveter, 1987

*Type and only genus*. *Rolfeia* Waterston, 1985.

*Distribution.* Carboniferous; United Kingdom.

*Emended diagnosis*. Limulina with transverse ridge nodes present on thoracetron; thoracetron with moveable lateral spines (after Lamsdell, 2016).

*Rolfeia* Waterston, 1985

(Fig. 1M)

*Type and only species*. *Rolfeia fouldensis* Waterston, 1985.

*Distribution.* Carboniferous; United Kingdom.

*Emended diagnosis*. Rolfeiid with lateral eyes positioned at apex of ophthalmic ridge that subsequently turns inwards; rounded thoracetron; opercular tergite distinct and produced into enlarged free lobes; thoracetron composed of six segments with enlarged pleural spines; moveable spines present, small.

Family PALEOLIMULIDAE Raymond, 1944

[=MORAVURIDAE Přibyl, 1967]

*Type genus*. *Paleolimulus* Dunbar, 1923.

*Included genera*. *Moravurus* Přibyl, 1967; *Norilimulus* gen. nov.; *Xaniopyramis* Siveter & Selden, 1987.

*Distribution.* Carboniferous–Permian; Canada, Czech Republic, Russia, United Kingdom, and United States.

*Emended diagnosis*. Limulina with pyramidal cheek node; interophthalmic ridges on prosomal shield; thoracetron with free lobes; moveable lateral spines present on thoracetron; transverse ridge nodes present on thoracetron (after Lamsdell, 2016).

*Moravurus* Přibyl, 1967.

*Type and only species*. *Moravurus rehori* Přibyl, 1967.

*Destribution.* Carboniferous; Czech Republic.

*Emended diagnosis*. Paleolimulid with semi-crescentic thoracetron; abaxial ridge present along length of thoracetron; pleura reduced.

*Norilimulus* gen. nov.

(Fig. 6A)

*Type and only species*. *Paleolimulus woodae* Lerner, Lucas & Mansky, 2016.

*Etymology.* From the Greek *νωρις* (early), referring to its occurrence as the oldest known paleolimulid, and -*limulus*, which has become something of a traditional epithet for fossil horseshoe crab species.

*Distribution.* Carboniferous; Canada.

*Diagnosis*. Paleolimulid with narrow genal spines; broad genal grooves ending in triangular-shaped termination; lacking interophthalmic ridges on prosomal shield; pleura of free lobe developed into a laterally extended distal spine; abaxial ridge present along length of thoracetron.

*Remarks*. *Norilimulus* is distinct from *Paleolimulus* in lacking interophthalmic ridges on its prosomal shield. The overall condition of the prosoma and thoracetron is more similar to *Moravurus* and *Xaniopyramis*, however the lack of transverse ridge nodes mark the genus as distinct from the other paleolimulids.

*Paleolimulus* Dunbar, 1923

(Fig. 1D)

*Type species*. *Prestwichia signata* Beecher, 1904 [=*Paleolimulus avitus* Dunbar, 1923].

*Included species. Paleolimulus* (?) *juresanensis* Chernyshev, 1933; *Paleolimulus kunguricus* Naugolnykh, 2017.

*Distribution.* Carboniferous–Permian; Russia and United States.

*Emended diagnosis*. Paleolimulid with interophthalmic ridges clustered around anterior of cardiac lobe; thoracetron markedly triangular.

*Remarks*. The species *Paleolimulus* (?) *juresanensis*, from the Permian of Russia, is known from a single poorly preserved specimen. While the general outline of the body is consistent with that of *Paleolimulus*, only the telson is preserved in any detail and the species is retained within the genus only with reservations.

*Xaniopyramis* Siveter & Selden, 1987

(Fig. 1C)

*Type and only species*. *Xaniopyramis linseyi* Siveter & Selden, 1987.

*Distribution.* Carboniferous; United Kingdom.

*Emended diagnosis*. Paleolimulid with narrow genal spines; fourth axial ridge of thoracetron extended abaxially into a transverse pleural ridge; abaxial ridge present along length of thoracetron; pleural spines reduced, moveable spines long and narrow.

Superfamily LIMULOIDEA Zittel, 1885

*Included taxa*. *Valloisella* Racheboeuf, 1992; Austrolimulidae Riek, 1955; Limulidae Zittel, 1885.

*Distribution.* Carboniferous–recent; worldwide.

*Emended diagnosis*. Limulina with thoracetron showing no lateral expression of individual tergites; moveable spines present on thoracetron; thoracetron with free lobes (after Lamsdell, 2016).

*Valloisella* Racheboeuf, 1992

*Type and only species*. *Valloisella lievinensis* Racheboeuf, 1992.

*Distribution.* Carboniferous; France and United Kingdom.

*Emended diagnosis*. Limuloid with elongate prosomal shield; genal spines elongate, narrow; opisthosomal axis hourglass-shaped, with carinate medial ridge running along its length; opisthosoma possessing six pairs of moveable spines.

**Table:** 

Family AUSTROLIMULIDAE Riek, 1955
[=DUBBOLIMULIDAE Pickett, 1984]

*Type genus*. *Austrolimulus* Riek, 1955.

*Included genera*. *Batracholimulus* gen. nov.; *Boeotiaspis* gen. nov.; *Dubbolimulus* Pickett, 1984; *Limulitella* Størmer, 1952 [= *Limulites* Schimper, 1853]; *Panduralimulus* Allen & Feldmann, 2005; *Psammolimulus* Lange, 1922; *Shpineviolimulus* Bicknell, Naugolnykh & Brougham, 2020; *Tasmaniolimulus* Bicknell, 2019; *Vaderlimulus* Lerner, Lucas & Lockley, 2017.

*Distribution.* Carboniferous–Triassic; Australia, France, Germany, Tunisia, Russia, Ukraine, and United States.

*Emended diagnosis*. Limuloidea with apodemal pits present on thoracetron; thoracetron lacking tergopleural fixed spines; posteriormost thoracetron tergopleurae swept back and elongated to form ‘swallowtail’; axis of thoracetron bearing dorsal keel (after Lamsdell, 2016).

**Table:** 

*Austrolimulus*Riek, 1955
(Fig. 1O)

*Type and only species*. *Austrolimulus fletcheri* Riek, 1955.

*Distribution.* Triassic; Australia.

*Emended diagnosis*. Austrolimulid with elongate, laterally oriented genal spines equal in length to the prosoma and thoracetron combined; enlarged, bulbous lateral eyes; ophthalmic ridges subdued anterior to lateral eyes; thoracetron smaller than prosomal shield, triangular without dorsal keel, lacking pleural spines or posterior ‘swallowtail’.

**Table:** 

*Boeotiaspis* gen. nov.
(Fig. 6C)

*Type and only species*. *Paleolimulus longispinus* Schram, 1979.

*Etymology.* Named after the Boeotian shield carried by warriors in ancient Greece, which the animal resembles with its broadly symmetrical prosoma and thoracetron. The suffix *aspis* meaning shield is applied, although the aspis was of a design distinct to the Boeotian shield.

*Distribution.* Carboniferous; United States.

*Diagnosis*. Austrolimulid with semi-circular prosomal shield; genal spines short but marginally splayed; thoracetron rounded; fixed pleural spines present; moveable spines greatly elongated; pretelsonic segment flanked by pair of elongating spines but not developed into ‘swallowtail’.

*Remarks*. ‘*Paleolimulus*’ *longispinus* is another species that has long been in need of a distinct generic assignment (Waterston, 1985; Anderson & Selden, 1997; Babcock & Merriam, 2000; Lamsdell, 2016; Lamsdell, 2020; Lerner, Lucas & Mansky, 2016; Lerner, Lucas & Lockley, 2017), a situation rectified here.

**Table:** 

*Batracholimulus* gen. nov.
(Fig. 6B)

*Type and only species*. *Paleolimulus fuchsbergensis* Hauschke & Wilde, 1987.

*Etymology.* From the Greek *β*ά*τραχ*o*ς* (frog), given the frog-like countenance afforded by the enlarged, posteriorly positioned lateral eyes, and -*limulus*.

*Distribution.* Triassic; Germany.

*Diagnosis*. Austrolimulid with short, splayed genal spines; enlarged, bulbous lateral eyes located posteriorly on prosomal shield; thoracetron triangular, gently curving after first few segments; lateral ridge running along fulcrum; small ‘swallowtail’ present.

*Remarks*. ‘*Paleolimulus*’ *fuchsbergensis* has been recognized to represent a novel genus of austrolimulid for several years (Anderson & Selden, 1997; Babcock & Merriam, 2000; Lamsdell, 2016; Lamsdell, 2020; Lerner, Lucas & Mansky, 2016; Lerner, Lucas & Lockley, 2017) and is finally elevated as such here.

*Dubbolimulus* Pickett, 1984

*Type and only species*. *Dubbolimulus peetae* Pickett, 1984.

*Distribution.* Triassic; Australia.

*Emended diagnosis*. Austrolimulid with semi-circular prosomal shield; prosomal shield shallow, splayed; enlarged, bulbous lateral eyes; ophthalmic ridges subdued anterior to lateral eyes; genal spines short; thoracetron small, approximately equal in total width to the ophthalmic ridges; thoracetron lateral margin smoothly curved, lacking pleural spines.

*Limulitella* Størmer, 1952

[= *Limulites* Schimper, 1853 (preoccupied)]

*Type species*. *Limulites bronni,* Schimper, 1853 [=*Limulus sandbergeri* Kirchner, 1923].

*Included species. Limulitella* (?) *liasokeuperensis* (Braun, 1860); *Limulitella tejraensis* Błazejowski et al., 2017; *Limulitella* (?) *volgensis* Ponomarenko, 1985.

*Distribution.* Triassic; France, Germany, Tunisia, and Russia.

*Emended diagnosis*. Austrolimulid with enlarged, bulbous lateral eyes; thoracetron subtriangular, showing no expression of individual tergites; lateral ridge running along fulcrum; abdominal segment not differentiated dorsally by groove.

*Remarks*. Both *Limulitella* (?) *volgensis* and *Limulitella* (?) *liasokeuperensis*, from the Triassic of Russia and Germany respectively, are of uncertain generic affinity, being fragmentarily preserved. The morphology of the cardiac lobe in both species may support an assignment to *Limulitella*, however this is not enough to assign them to the genus without reservation.

*Panduralimulus* Allen & Feldmann, 2005

*Type and only species*. *Panduralimulus babcocki* Allen & Feldmann, 2005.

*Distribution.* Permian; United States.

*Emended diagnosis*. Austrolimulid with violin-shaped cardiac lobe; ophthalmic ridge parabolic, smoothly curving; thoracteron free lobes pronounced, posteriorly directed; thoracetron lacking pleural spines except posteriormost pair, which are elongated.

*Psammolimulus* Lange, 1922

*Type and only species*. *Psammolimulus gottingensis* Lange, 1922.

*Distribution.* Triassic; Germany.

*Emended diagnosis*. Austrolimulid with elongated genal spines extending to posterior of thoracetron; enlarged, bulbous lateral eyes; thoracetron trapezoidal, showing no expression of individual tergites; free lobe produced into distinct cornua; moveable spines short, robust; lacking pleural spines except for posteriormost pair, which are enlarged.

*Shpineviolimulus* Bicknell, Lustri & Brougham, 2019

*Type and only species*. *Paleolimulus jakovlevi* Glushenko & Ivanov, 1961.

*Distribution.* Permian; Ukraine.

*Emended diagnosis*. Austrolimulid with semi-circular prosomal shield; genal spines short but marginally splayed; occipital lobes inflated, extend to tips of genal spines; fixed pleural spines absent; pretelsonic segment flanked by pair of elongating spines but not developed into ‘swallowtail’.

*Tasmaniolimulus* Bicknell, 2019

*Type and only species*. *Tasmaniolimulus patersoni* Bicknell, 2019.

*Distribution.* Permian; Australia.

*Emended diagnosis*. Austrolimulid with genal spines extending posteriorly to posterior margin of thoracetron without substantial splay; ophthalmic ridges forming prominent ‘m’ shape; thoracetron smaller than cephalothorax.

*Vaderlimulus* Lerner, Lucas & Lockley, 2017

*Type and only species*. *Vaderlimulus tricki* Lerner, Lucas & Lockley, 2017.

*Distribution.* Triassic; United States.

*Emended diagnosis*. Austrolimulid with semicircular prosoma; enlarged, bulbous lateral eyes; ophthalmic ridges subdued anterior to lateral eyes; large posterolaterally directed genal spines terminate approximately in-line with the telson boss; thoracetron length slightly more than half that of the prosoma, lacking dorsal keel; free lobes laterally extend to a distance that is approximately equal to thoracetron length; pleural spines absent except for posterior pair which are short and broad; telson at least equal in length to the remainder of the body.

**Table:** 

Family LIMULIDAE Zittel, 1885
[=MESOLIMULIDAE Størmer, 1952; =HETEROLIMULIDAE Vía Boada & De Villalta, 1966]

*Type genus*. *Limulus* Müller, 1785 [=*Monoculus* Linnaeus, 1758; =*Xiphosura* Gronovius, 1764].

*Included genera*. *Allolimulus* gen. nov.; *Carcinoscorpius* Pocock, 1902; *Casterolimulus* Holland, Erickson & O’Brien, 1975; *Crenatolimulus* Feldmann et al., 2011; *Heterolimulus* Vía Boada & De Villalta, 1966; *Keuperlimulus* gen. nov.; *Mesolimulus* Størmer, 1952; *Tachypleus* Leach, 1819; *Tarracolimulus* Romero & Vía Boada, 1977; *Victalimulus* Riek & Gill, 1971; *Volanalimulus* gen. nov.; *Yunnanolimulus* Zhang et al., 2009.

*Distribution.* Triassic–recent; Australia, Bangladesh, China, France, Germany, India, Indonesia, Japan, Lebanon, Madagascar, Malaysia, Morocco, Myanmar, Philippines, Poland, Russia, Singapore, Spain, Taiwan, Thailand, United Kingdom, United States, and Vietnam.

*Emended diagnosis*. Limuloidea with thoracetron showing no expression of individual tergites; axis of thoracetron bearing dorsal keel; apodemal pits sometimes present (after Lamsdell, 2016).

**Table:** 

*Allolimulus* gen. nov.
(Fig. 5F)

*Type and only species*. *Limulus woodwardi* Watson, 1909.

*Etymology.* The name translates as “other *Limulus*”, reflecting the initial misidentification of the type species as a species of *Limulus*.

*Distribution.* Jurassic; United Kingdom.

*Diagnosis*. Limulid with broad, shallow prosomal shield; lateral eyes located on posterior third of prosomal shield; cardiac lobe with well-defined median ridge with rounded cross section, lacking protuberances or spines; cardiac lobe flanked by deep axial furrows; genal spines short, with genal facet expanding distally.

*Remarks*. *Allolimulus* exhibits close affinity to the Cretaceous limulids *Casterolimulus* and *Victalimulus*, with the cardiac lobe flanked by deep axial furrows and bearing a median ridge with rounded cross section.

**Table:** 

*Carcinoscorpius*Pocock, 1902

*Type and only species*. *Limulus rotundicauda* Latreille, 1802.

*Distribution.* Recent; Bangladesh, India, Indonesia, Malaysia, Myanmar, Singapore, and Thailand.

*Emended diagnosis*. Limulid with shallow, semi-circular prosomal shield; genal groove terminating at proximal third of genal spine; small ophthalmic spines present at posterior of ophthalmic ridges; pleura of free lobe reduced, terminating before thoracetron margin; posteriormost fixed pleural spines on thoracetron broad, with the distal angle of the spine equal to or greater than 90 degrees; telson with ventral groove.

**Table:** 

*Casterolimulus*Holland, Erickson & O’Brien, 1975

*Type and only species*. *Casterolimulus kletti* Holland, Erickson & O’Brien, 1975.

*Distribution.* Cretaceous; United States.

*Emended diagnosis*. Limulid with shallow prosomal shield; ophthalmic ridges curving medially towards the cardiac lobe anteriorly but becoming effaced before reaching it; cardiac lobe with well-defined median ridge with rounded cross section, lacking protuberances or spines; width of cardiac lobe less than one third of cardiac lobe length; cardiac lobe flanked by deep axial furrows, angled obliquely toward the anterior end of the median ridge; margins of genal spines subparallel to central axis, becoming laterally more oblique toward their tips.

**Table:** 

*Crenatolimulus*Feldmann et al., 2011

*Type species*. *Crenatolimulus paluxyensis* Feldmann et al., 2011.

*Included species. Crenatolimulus darwini* (Kin & Błażejowski, 2014) comb. nov.

*Distribution.* Jurassic–Cretaceous; Poland and United States.

*Emended diagnosis*. Limulid with highly vaulted prosomal shield; posterior rim of prosomal shield prominent and depressed; rectangular cardiac lobe; genal groove terminating at proximal third of genal spine; pleura of free lobe reduced, terminating before thoracetron margin; thoracetron with scalloped lateral margins and crenulate flanks.

*Remarks*. ‘*Limulus*’ *darwini*, from the Jurassic Kcynia Formation of Poland, has never been resolved explicitly as a member of *Limulus* in any phylogenetic analysis, instead forming a polytomy with *Limulus* and *Crenatolimulus* (Lamsdell, 2016; Lamsdell, 2020). An undescribed species of *Crenatolimulus* has been documented as co-occurring with ‘*Limulus*’ *darwini* (Kin et al., 2013; Błazejowski, 2015; Błazejowski et al., 2019), and the two have been considered to be conspecific previously (Tashman, 2014). The holotype of ‘*Limulus*’ *darwini* (ZPAL X.10-BXA) actually exhibits scalloping on the left lateral margin of the thoracetron (the right margin is not preserved), confirming both species of limulid in the Kcynia Formation to be conspecific and necessitating its transferal to *Crenatolimulus*.

**Table:** 

*Heterolimulus*Vía Boada & De Villalta, 1966

*Type and only species*. *Heterolimulus gadeai* Vía Boada & De Villalta, 1966.

*Distribution.* Triassic; Spain.

*Emended diagnosis*. Limulid with genal groove terminating at proximal third of genal spine; pleura of free lobe reduced, terminating before thoracetron margin; thoracetron width constant for anterior half; posteriormost fixed pleural spines on thoracetron broad, with the distal angle of the spine equal to or greater than 90 degrees; lateral ridge running along fulcrum; telson with ventral groove.

*Remarks*. *Heterolimulus* has previously been considered to be a synonym of *Tachypleus* (Diedrich, 2011; Lamsdell & McKenzie, 2015; Lamsdell, 2016), but is here shown to be a distinct genus, representing the sister taxon to a clade comprising the genera *Tachypleus* and *Carcinoscorpius*.

**Table:** 

*Keuperlimulus* gen. nov.
(Fig. 6D)

*Type and only species*. *Limulus vicensis* Bleicher, 1897

*Etymology.* Named for the Keuper lithostratigraphic unit, which comprises the Carnian–Norian in Central Europe, from which the type species is found.

*Distribution.* Triassic; France.

*Diagnosis*. Limulid with broad, semi-circular prosomal shield; ophthalmic ridges converging steadily anteriorly; cardiac lobe elongated, extending to anterior third of prosomal shield; cardiac lobe flanked by deep axial furrows; pleura of free lobe reduced, terminating before thoracetron margin; thoracetron lacking axial nodes.

*Remarks*. Another species previously assigned to the wastebasket taxon of *Limulitella*. While the type species of *Limulitella* resolves as an austrolimulid, *Keuperlimulus vicensis* is a limulid with close affinities to *Mesolimulus* and the clade including *Victalimulus*.

**Table:** 

*Limulus*Müller, 1785
[=*Monoculus*Linnaeus, 1758; =*Xiphosura*Gronovius, 1764]

*Type species*. *Monoculus polyphemus* Linnaeus, 1758 [=*Limulus cyclops* Fabricius, 1793; =*Limulus occidentalis* Lamarck, 1801; =*Limulus albus* Bosc, 1802; =*Limulus sowerbii* Leach, 1815; =*Limulus americanus* Leach, 1819].

*Included species. Limulus coffini* Reeside & Harris, 1952; ‘*Limulus*’ *priscus* Münster, 1839.

*Distribution.* Cretaceous–recent; United States.

*Emended diagnosis*. Limulid with heavily domed prosomal shield; rectangular cardiac lobe; genal groove terminating at proximal third of genal spine; pleura of free lobe reduced, terminating before thoracetron margin; free lobe folded back on itself and expanded anteriorly, resulting in wedge-shaped morphology; appendage III not modified into claspers in males.

*Remarks.* ‘*Limulus*’ *priscus* is poorly preserved and displays no diagnostic characteristics. The thoracetron appears much smaller than the prosoma and the species almost certainly does not belong within *Limulus*, however it is currently impossible to assign it to any other genus with any confidence and the species may be considered a *nomen dubium*.

**Table:** 

*Mesolimulus*Størmer, 1952
(Figs. 1K, 1L)

*Type species*. *Limulus walchi* Desmarest, 1822 [=*Limulus brevicauda* Münster *in* Van Der Hoeven, 1838; =*Limulus brevispina* Münster *in* Van Der Hoeven, 1838; =*Limulus intermedius* Münster *in* Van Der Hoeven, 1838; =*Limulus ornatus* Münster *in* Van Der Hoeven, 1838; =*Limulus sulcatus* Münster *in* Van Der Hoeven, 1838; =*Limulus giganteus* Münster, 1840].

*Included species. Mesolimulus crispelli* Vía, 1987; *Mesolimulus sibiricus* Ponomarenko, 1985; *Mesolimulus tafraoutensis* Lamsdell et al., 2020.

*Distribution.* Triassic–Cretaceous; Germany, Morocco, Spain, and Russia.

*Emended diagnosis*. Limulid with prosoma wider than long; cardiac lobe narrow with scalloped margins, parallel sided with keel developed into median cardiac ridge with rounded cross section, flanked by deep axial furrows; thoracetron wider than long, bearing apodemal pits; pleura of free lobe reduced, terminating before thoracetron margin; thoracetron margins bearing five moveable and six fixed spines; lateral ridge running along fulcrum.

**Table:** 

*Tachypleus*Leach, 1819

*Type species*. *Limulus gigas* Müller, 1785 [=*Limulus heterodactylus* Latreille, 1802; =*Limulus moluccanus* Latreille, 1802; =*Limulus viriscens* Latreille, 1806; =*Limulus latreillii* Leach, 1819; =*Limulus macleaii* Leach, 1819; =*Tachypleus hoeveni* Pocock, 1902].

*Included species. Tachypleus tridentatus* Leach, 1819 [=*Limulus longispina* Van Der Hoeven, 1838]; *Tachypleus syriacus* Woodward, 1879; *Tachypleus decheni* Zinken, 1862.

*Distribution.* Cretaceous–recent; Bangladesh, China, Germany, India, Indonesia, Japan, Lebanon, Malaysia, Myanmar, Philippines, Singapore, Taiwan, Thailand, and Vietnam.

*Emended diagnosis*. Limulid with lateral eyes positioned at apex of ophthalmic ridge that subsequently turns inwards; genal groove terminating at proximal third of genal spine; small ophthalmic spines present at posterior of ophthalmic ridges; axial portion of free lobe segment of thoracetron bearing large spine; posteriormost fixed pleural spines on thoracetron broad, with the distal angle of the spine equal to or greater than 90 degrees; telson with ventral groove.

**Table:** 

*Tarracolimulus*Romero & Vía Boada, 1977

*Type and only species*. *Tarracolimulus reiki* Romero & Vía Boada, 1977.

*Distribution.* Triassic; Spain.

*Emended diagnosis*. Limulid with relatively short genal spines; ophthalmic ridges and cardiac lobe pronounced, ophthalmic ridges effaced anterior to cardiac lobe; interophthalmic ridges on prosomal shield; thoracetron triangular in shape, narrowing evenly posteriorly; pleura of free lobe reduced, terminating before thoracetron margin; pleural spines present, angled posteriorly; six pairs of moveable spines present.

**Table:** 

*Victalimulus*Riek & Gill, 1971
(Fig. 1N)

*Type and only species*. *Victalimulus mcqueeni* Riek & Gill, 1971.

*Distribution.* Cretaceous; Australia.

*Emended diagnosis*. Limulid with cardiac lobe bearing well-defined median ridge with rounded cross section, bearing three protuberances or spines; width of cardiac lobe less than one third of cardiac lobe length; cardiac lobe flanked by deep axial furrows, converging anteriorly; ophthalmic ridge defined for a moderate distance anterior to the lateral eye, not converging strongly anteriorly; outer margin of genal spine parallel to median axis of body; thoracetron with strongly convex margins, free lobe distinct; pleura of free lobe reduced, terminating before thoracetron margin; apodemal pits present; marginal spines long, directed posteriorly.

**Table:** 

*Volanalimulus* gen. nov.
(Fig. 6E)

*Type and only species*. *Volanalimulus madagascarensis* sp. nov.

*Etymology.* The name is derived from the Malagasy word *volana*, meaning moon, in reference to the broad crescentic shape of the prosomal shield.

*Distribution.* Triassic; Madagascar.

*Diagnosis*. Limulid with genal groove terminating at proximal third of genal spine; pleura of free lobe reduced, terminating before thoracetron margin; thoracetron bearing longitudinal ridges along fulcrum; apodemal pits present; posteriormost fixed pleural spines on thoracetron broad, with the distal angle of the spine equal to or greater than 90 degrees.

*Remarks*. Hauschke, Wilde & Brauckmann (2004) described a limulid from the Lower Triassic of Madagascar, comparing the species to *Limulitella* but leaving it in open nomenclature. While one of the two available specimens is poorly preserved, the other displays details of the external dorsal surface of the prosoma, thoracetron and telson and possesses a unique suite of characteristics that show it to be a distinct species. Furthermore, phylogenetic analysis resolves the new species as a novel genus.

**Table:** 

*Volanalimulus madagascarensis* sp. nov.
(Fig. 6E)
cf. *Limulitella* sp. Hauschke, Wilde & Brauckmann, 2004 Figs. 2 and 3

*Holotype.* TUCP Ch 5, almost complete specimen comprising prosoma, thoracetron and proximal portion of telson in dorsal view.

*Additional material.* Paratype, MSNM No. I 11170, part and counterpart of prosoma, thoracetron and telson in ventral view. Possibly exhibiting soft tissue preservation, but details overall lacking.

*Etymology.* Named after Madagascar, the region from which it is found.

*Distribution.* Triassic; Madagascar.

*Diagnosis.* As for genus.

*Description.* See Hauschke, Wilde & Brauckmann (2004) for a full description of the specimens.

**Table:** 

*Yunnanolimulus*Zhang et al., 2009

*Type species*. *Yunnanolimulus luopingensis* Zhang et al., 2009.

*Included species. Yunnanolimulus henkeli* (Fritsch, 1906) comb. nov.

*Distribution.* Triassic; China and Germany.

*Emended diagnosis*. Limulid with gently vaulted semi-circular prosomal shield; cardiac lobe tapering gradually forward; ophthalmic ridges distinct, not meeting in front of cardiac lobe; genal spines triangular, posteriorly directed; thoracetron subtriangular, slightly wider than cardiac lobe, tapering backward gradually; axis distinct, with median keel; subaxial ridges running along length of thoracetron; transverse ridge nodes present on thoracetron; six pairs of moveable spines present; abdominal segment demarcated dorsally by groove; telson long, triangular in cross-section.

*Remarks. Yunnanolimulus henkeli* has previously been assigned to *Limulitella*, however phylogenetic analysis has retrieved it as the sister species to *Yunnanolimulus luopingensis*. The available characteristics of *Y. henkeli*, namely the gradually tapering cardiac lobe, posteriorly directed triangular genal spines, subtriangular thoracetron, and subaxial ridges all correspond well to *Yunnanolimulus*. As such, the species is transferred to the genus herein.

**Table:** 

*Incertae sedis*
*Albalimulus*Bicknell & Pates, 2019

*Type and only species*. *Albalimulus bottoni* Bicknell & Pates, 2019.

*Distribution.* Carboniferous; United Kingdom.

*Emended diagnosis*. Xiphosurid with pustulose cuticular ornament.

*Remarks*. *Albalimulus bottoni* is known from a single specimen preserved in part and counterpart. The available material shows only the general outline of the animal with a number of deformation wrinkles on its surface, some of which may represent structures such as lateral eyes and ophthalmic ridges. The most distinctive feature of the taxon is the patchily pustular ornamentation located on parts of the thoracetron and prosoma. Pustulose ornamentation is otherwise known only from *Belinurus pustulosus*, although it is worth noting that the majority of fossil horseshoe crabs do not preserve the cuticle, and in those that do it is finely granular (Lamsdell et al., 2020). Ornamentation is known to remain at a relatively constant size during the ontogeny of eurypterids (Lamsdell & Selden, 2013) and given the exceedingly small size of *Albalimulus bottoni* (the holotype being only 12.5 mm long) it is possible that the pustules are actually granules on a juvenile individual. Other traits of *Albalimulus bottoni* point towards its being a juvenile; the broad-based telson, short genal spines, and general lack of dorsal features are all reminiscent of modern xiphosurids during the first six or seven molts (Lamsdell, 2020). *Albalimulus* being such an early instar places it in the same position as *Xiphosuroides*, as it is likely that it may represent a synonym of an existing genus of Carboniferous horseshoe crab. *Rolfeia* (which has a body length of at least 60 mm) is also known from the Tournaisian of Scotland and is a potential candidate, however no other horseshoe crabs are currently known from the Ballagan Formation alongside *Albalimulus* and so no synonymy is suggested at this time. Like *Xiphosuroides*, *Albalimulus* should be considered *incertae sedis*; however, the lack of morphological features precludes its assigned to any group beyond Xiphosurida.

*Sloveniolimulus* Bicknell et al., 2019

*Type and only species*. *Sloveniolimulus rudkini* Bicknell et al., 2019.

*Distribution.* Triassic; Slovenia.

*Emended diagnosis*. Xiphosurid with semi-circular prosomal shield; genal spines indented, deflected away from thoracetron.

*Remarks*. *Sloveniolimulus* is known from only a single, poorly preserved specimen displaying little more than the outline of the animal. The establishment of a new genus and species was justified based on the deflection of the genal spines away from the thoracetron, however the pliability of limulid carapaces post mortem is well documented (Babcock & Chang, 1997; Babcock, Merriam & West, 2000) and the utility of genal spine angle as a diagnostic trait in a specimen not preserving any other identifiable features is suspect. As such, *Sloveniolimulus rudkini* can be considered at best as *incertae sedis* within Xiphosurida, and might even need to be classified as *nomen dubium* unless additional, better preserved material comes to light.

## Discussion

Macroevolutionary and macroecological studies of Xiphosurida have recognized ecological (Lamsdell, 2016) and heterochronic (Lamsdell, 2020) trends within the group, with belinurines and austrolimulids occupying non-marine environments and exhibiting concerted shifts to paedomorphic and peramorphic modes of evolution respectively. These trends hold up to the addition of more taxa into the analysis, including the recently described belinurine *Stilpnocephalus* and austrolimulid *Tasmaniolimulus*, both of which are known from non-marine strata. While *Tasmaniolimulus* closely resembles other early austrolimulids (Bicknell, 2019), *Stilpnocephalus* is at first glance a very aberrant belinurine, being larger than the other species in the group and with a highly effaced prosomal shield (Selden, Simonetto & Marsiglio, 2019). Evaluating *Stilpnocephalus* within a phylogenetic context demonstrates that its unusual morphology is a continuation of the trends observed in *Liomesaspis* and *Alanops*, which display a general decrease in the size of the lateral eyes, reduction of the genal and ophthalmic spines, and progressive effacement of the ophthalmic ridges and cardiac furrows (Racheboeuf, Vannier & Anderson, 2002).

Previous analyses of xiphosuran phylogeny have suggested that synziphosurines, previously considered stem Xiphosura, are a polyphyletic assemblage of stem euchelicerates, Xiphosura, and species more closely related to eurypterids and arachnids (Lamsdell, 2013; Lamsdell, 2016). Subsequent discoveries of synziphosurines with thirteen opisthosomal segments (Lamsdell et al., 2015) and a metastoma (Selden, Lamsdell & Liu, 2015), chasmataspidid and eurypterid characteristics, have reinforced the hypothesis that most synziphosurines do not resolve within the clade Xiphosura and this revised understanding of their relationships continues to be supported here. This results in a 61 million year gap in the xiphosuran fossil record between their first occurrences in the Ordovician and their next subsequent record in the Upper Devonian (Lamsdell, in press). Xiphosuran diversity peaks in the Carboniferous with the radiation of the belinurines; of the 45 known Carboniferous species, 37 belong within Belinurina. The number of recognised valid belinurine species has decreased over the last couple of decades, with twelve species synonymized with *Euproops danae* by Anderson (1994). Subsequently, Haug et al. (2012) have suggested that several other *Euproops* species may be synonyms, representing different ontogenetic stages. Most recently, some small specimens assigned to *Belinurus* have also been suggested to be juvenile instars of *Euproops* (Haug & Haug, 2020). Differentiating between juvenile stages and distinct species is further complicated by heterochronic trends within Belinurina (Haug & Rötzer, 2018; Lamsdell, 2020), and further studies of individual species ontogeny will likely be required to provide a final resolution to the issue. Including multiple ontogenetic stages of a single species within a phylogeny has been demonstrated to destabilise the resulting tree topology (Lamsdell & Selden, 2013; Lamsdell & Selden, 2015), with juveniles resolving stemward relative to the adults. This stemward slippage is also associated with the collapsing of internal phylogenetic nodes into polytomies, due to the mosaic nature of character change and the resulting conflicts in character states between erroneously included juvenile ‘ontospecies’ and valid species (Lamsdell & Selden, 2013). The high degree of resolution in the current phylogeny would suggest that the number of ‘ontospecies’ included, if any, is at a minimum, while most species are known from individuals of approximately equivalent size (with some notable exceptions, such as *Albalimulus*). This suggests that, while individual specimens may have been mis-assigned (as identified by Haug & Haug, 2020), the genera recognized herein are valid and the diversity of Belinurina, though maybe not as high as currently projected, is still greater than that of other xiphosurid clades.

## Conclusions

Xiphosuran phylogenetic relationships have proven robust to the addition of new taxa and analysis under different optimality criteria, with tree topology concordant between Bayesian and parsimony analysis, over seven years of study and with the incorporation of 120 additional species since the initial iteration of the current phylogenetic matrix. The long stability of the current phylogenetic hypothesis indicates its suitability in forming the basis of an updated taxonomy of Xiphosura. Bringing xiphosuran taxonomy in line with our phylogenetic understanding of the group ensures that the higher taxa within the group represent true biological units (clades), which is necessary for ensuring the accuracy of meta analyses that rely upon the hierarchical taxonomic framework to infer organismal relationships (Lamsdell et al., 2017). This taxonomic update also lays the groundwork for the eventual revision of the chelicerate volume of the *Treatise on Invertebrate Paleontology*. The updated taxonomy and phylogenetic framework will facilitate exploration of fundamental questions surrounding horseshoe crab evolution, including why the modern species exhibit their disjunct geographic distribution, whether horseshoe crabs genuinely exhibit constant slow rates of morphological evolution over their evolutionary history as assumed by their frequent categorization as ‘living fossils’, and how a lineage with only a handful of species is likely to respond to climate perturbations in the future.

##  Supplemental Information

10.7717/peerj.10431/supp-1 Supplemental Information 1 Phylogenetic data matrix of Xiphosura in NEXUS file format Phylogenetic character matrix utilised in the study. Saved in NEXUS format.

10.7717/peerj.10431/supp-2 Supplemental Information 2 Parsimony phylogenetic tree Maximum parsimony strict consensus of 6 most parsimonious trees. Values beneath nodes are Bootstrap/Bremer/Jacknife support values.

## Institutional abbreviations

AM: Australian Museum, Sydney, New South Wales, Australia
BGS: British Geological Survey, Nottingham, England, UK
FMNH: Field Museum of Natural History, Chicago, Illinois, USA
MAN: Muséum-Aquarium de Nancy, Lorraine, France
MM: Manitoba Museum, Winnipeg, Manitoba, Canada
MMUP: Manchester Museum, Manchester, England, UK
MNHN: Museum National d’Histoire Naturelle, Paris, France
MSNM: Museo Civico di Storia Naturale di Milano, Milan, Italy
NMS: National Museums of Scotland, Edinburgh, Scotland, UK
NMV: Museums Victoria, Carlton, Victoria, Australia
NMW: National Museum of Wales, Cardiff, Wales, UK
NSM: Nova Scotia Museum, Halifax, Nova Scotia, Canada
OUM: Oxford University Museum of Natural History, Oxford, England, UK
ROM: Royal Ontario Museum, Toronto, Ontario, Canada
SMF: Senckenberg Forschungsinstitut und Naturmuseum, Frankfurt am Main, Germany
TUCP: Technische Universität Institut für Geologie und Paläontologie, Clausthal-Zellerfeld, Germany
YPM: Yale Peabody Museum, New Haven, Connecticut, USA

## Additional Information and Declarations

### Data Availability

The following information was supplied regarding data availability: Data for this study are available at MorphoBank P3497 and in the Supplementary Files.
https://morphobank.org/index.php/Projects/ProjectOverview/project_id/3497. Specimens figured in the manuscript are accessioned at the Australian Museum, Sydney, New South Wales, Australia (AM F68969, AM F38274); British Geological Survey, Nottingham, England, UK (BGS GSM49362); Field Museum of Natural History, Chicago, Illinois, USA (FMNH PE.56581); Muséum-Aquarium de Nancy, Lorraine, France (MAN 8240); Manitoba Museum, Winnipeg, Manitoba, Canada (MM I-4583); Manchester Museum, Manchester, England, UK (MMUP L.8627); Museum National d’Histoire Naturelle, Paris, France (MNHN.F.A33516); Museo Civico di Storia Naturale di Milano, Milan, Italy (MSNM i26844, MSNM i9352); National Museums of Scotland, Edinburgh, Scotland, UK (NMS G.2007.271.A, NMS G.1984.67.1B); Museums Victoria, Carlton, Victoria, Australia (NMV P22410B); National Museum of Wales, Cardiff, Wales, UK (NMW 29.197.G3, NMW 29.198.G1); Nova Scotia Museum, Halifax, Nova Scotia, Canada (NSM 005GF045.374); Oxford University Museum of Natural History, Oxford, England, UK (OUM E.03994); Royal Ontario Museum, Toronto, Ontario, Canada (ROM IP45851); Senckenberg Forschungsinstitut und Naturmuseum, Frankfurt am Main, Germany (SMF VII I 311); Technische Universität Institut für Geologie und Paläontologie, Clausthal-Zellerfeld, Germany (TUCP Ch.5); and Yale Peabody Museum, New Haven, Connecticut, USA (YPM IP 026324, YPM IP 000125, YPM IP 428963, YPM IZ 055605, YPM IZ 070174, YPM IZ 055595, YPM IZ 055574, YPM IZ 055578, YPM IZ 055570, YPM IZ 055581, YPM IZ 055576, YPM IZ 070174).

### New Species Registration

The following information was supplied regarding the registration of a newly described species: Publication LSID: urn:lsid:zoobank.org:pub:3653AFDA-318D-4A1D-9E24-4F9F3D30C424 *Allolimulus*: urn:lsid:zoobank.org:act:236EACF3-BC00-4F40-9D3A-D595983FDD9F *Andersoniella*: urn:lsid:zoobank.org:act:71A1F53C-1C4E-4D3C-B6E0-F9144141D617 *Batracholimulus*: urn:lsid:zoobank.org:act:65347E99-A7FF-4426-9F97-E5CB78A41ADD *Boeotiaspis*: urn:lsid:zoobank.org:act:03CDF266-1553-486D-8310-61E585D2C81F *Keuperlimulus*: urn:lsid:zoobank.org:act:10A6B1A6-7C1C-4767-B16B-76DAF1FE3938 *Macrobelinurus*: urn:lsid:zoobank.org:act:DEAE80E8-F135-43FE-A19A-24BA7CCDF603 *Norilimulus*: urn:lsid:zoobank.org:act:177CF385-D4E4-442B-9E96-D0C896B6B86C *Parabelinurus*: urn:lsid:zoobank.org:act:5F50A91F-ABAE-4BE8-924C-09EEC51B1F8C *Volanalimulus*: urn:lsid:zoobank.org:act:AA6B8DC3-9FBB-4ED9-91DA-DAA797AC54A9 *Volanalimulus madagascarensis*: urn:lsid:zoobank.org:act:4AB258D3-3487-4CA6-9C59-D29CDBA0F5FB.
