# Supplementary figures and images for: The phylogeny and systematics of Xiphosura
Source: PeerJ. 2020 Dec 4;8:e10431. doi: 10.7717/peerj.10431 (PMC7720731; doi:10.7717/peerj.10431)

Values beneath nodes are *Bootstrap*/**Bremer**/Jackknife support values.

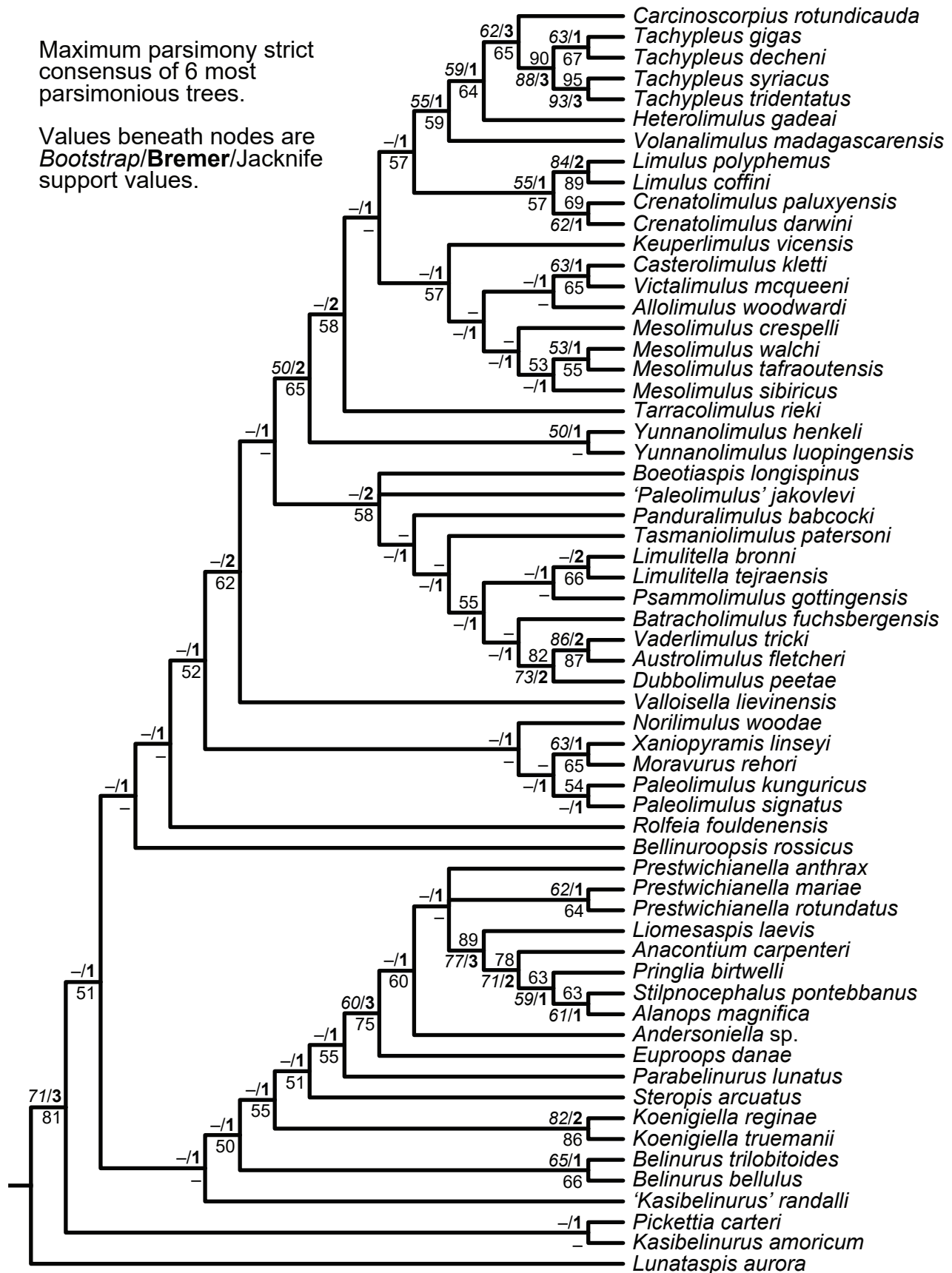

Supplement: Supplemental Information 2 — Maximum parsimony strict consensus of 6 most parsimonious trees. Values beneath nodes are Bootstrap/Bremer/Jacknife support values. [file peerj-08-10431-s002.pdf]
